# Optogenetic control of nuclear protein export

**DOI:** 10.1038/ncomms10624

**Published:** 2016-02-08

**Authors:** Dominik Niopek, Pierre Wehler, Julia Roensch, Roland Eils, Barbara Di Ventura

**Affiliations:** 1 Department of Theoretical Bioinformatics, German Cancer Research Center (DKFZ), Im Neuenheimer Feld 280, 69120 Heidelberg, Germany; 2 Department of Bioinformatics and Functional Genomics, Synthetic Biology Group, Institute for Pharmacy and Biotechnology (IPMB), University of Heidelberg, Im Neuenheimer Feld 364, 69120 Heidelberg, Germany; 3 Center for Quantitative Analysis of Molecular and Cellular Biosystems (BioQuant), University of Heidelberg, Im Neuenheimer Feld 267, 69120 Heidelberg, Germany

## Abstract

Active nucleocytoplasmic transport is a key mechanism underlying protein regulation in eukaryotes. While nuclear protein import can be controlled in space and time with a portfolio of optogenetic tools, protein export has not been tackled so far. Here we present a light-inducible nuclear export system (LEXY) based on a single, genetically encoded tag, which enables precise spatiotemporal control over the export of tagged proteins. A constitutively nuclear, chromatin-anchored LEXY variant expands the method towards light inhibition of endogenous protein export by sequestering cellular CRM1 receptors. We showcase the utility of LEXY for cell biology applications by regulating a synthetic repressor as well as human p53 transcriptional activity with light. LEXY is a powerful addition to the optogenetic toolbox, allowing various novel applications in synthetic and cell biology.

## Abstract (web summary)

Light-inducible control of protein subcellular localization holds great promise for synthetic biology applications and insights into basic cell biology. Here the authors develop a genetically-encoded light-inducible nuclear export system and apply it to a synthetic repressor and p53 transcriptional activity.

## Graphical abstract

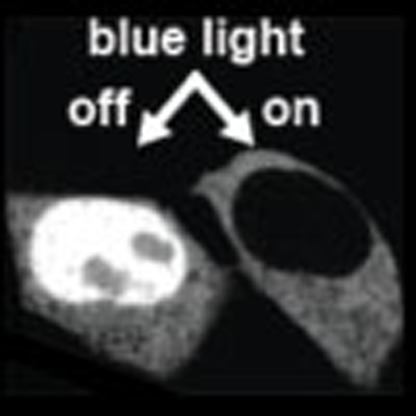

Active nucleocytoplasmic transport controls the localization and spatiotemporal dynamics of proteins in eukaryotes, thereby governing essential cellular processes including gene expression, cell division and apoptosis. Regulation of protein import and export is achieved mainly by masking and unmasking of nuclear import and nuclear export signals (NLSs and NESs) directly located within the polypeptide or by binding and unbinding to NLS- and NES-bearing partners^1^.

Optogenetic tools that enable controlling with light the nuclear import of tagged proteins in mammalian cells and yeast have been reported^2^,^3^,^4^,^5^,^6^, but no optogenetic tools are yet available to directly control protein export. However, such a tool would have enormous application potential, for example, for regulating the activity of nuclear or cytoplasmic signalling molecules, and would complement the existing optogenetic toolset for control of nuclear import^2^,^3^,^4^,^5^,^6^, protein dimerization^7^ and oligomerization^8^,^9^, membrane recruitment^10^ and organelle transport and positioning^11^.

Here we present LEXY, a blue light-induced nuclear export system enabling dynamic and spatial control over nuclear protein export. We show fast and fully reversible nuclear export of LEXY-tagged proteins of diverse nature and origin in various cell lines. A chromatin-anchored LEXY variant mediates light-inducible sequestration of cellular CRM1, the primary nuclear export receptor, thereby allowing inhibition of endogenous nuclear export. To demonstrate the utility of LEXY for applications in synthetic and cell biology, we regulate synthetic repressors as well as the transcriptional activity of human p53 with light.

## Results

### LEXY engineering and characterization

**Figure 1:**
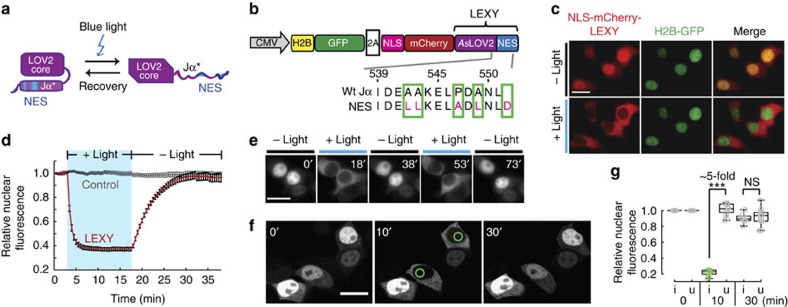
Engineering and characterization of LEXY, a light-inducible nuclear export system. (**a**) Schematic of LEXY function. Blue light irradiation releases the photocaged NES from the *As*LOV2 core, thereby inducing nuclear export. (**b**) Schematic of a construct encoding NLS-mCherry-LEXY and a H2B-GFP nuclear marker. NLS, cMyc^P1A^ NLS; 2A, T2A peptide. Green boxes indicate amino-acid residues altered as compared with the wild-type *As*LOV2 Jα helix (Wt Jα). Numbers show the position of the corresponding residues in the full-length *As*LOV2 domain. (**c**) Fluorescence images of HEK 293T cells transiently transfected with the construct in **b** before and after 15 min of pulsatile blue light irradiation. Scale bar, 20μm. (**d**) Relative nuclear fluorescence of HEK 293T expressing NLS-mCherry-LEXY (LEXY) or NLS-mCherry-*As*LOV2 (control) over time. Cells were incubated in the dark for 3 min before blue light irradiation for 15 min followed by a 20-min dark-recovery phase (mean±s.e.m., *n*=22 cells from 3 independent experiments). (**e**) Fluorescence images of NLS-mCherry-LEXY-expressing cells undergoing repeated illumination and recovery cycles. Scale bar, 20μm. (**f**) Fluorescence images of cells expressing NLS-mCherry-LEXY individually irradiated. Green circles depict regions scanned with a blue laser beam for 10 min following 20 min of dark recovery. Scale bar, 20μm. (**g**) Quantification of the relative nuclear fluorescence of individually irradiated cells (i) and uninduced control cells (u) at the indicated time points. Data represent box plots and individual data points, *n*=8 induced cells and 13 uninduced cells from 3 independent experiments; ***P=1.69 × 10^−17^ by Welch's *t*-test. NS, not significant.

LEXY consists of an engineered LOV2 domain from *Avena sativa* phototropin-1 (*As*LOV2), in which the C-terminal Jα helix was converted into an artificial NES. In the dark, the NES is tightly packed against the *As*LOV2 core and is thus inactive (Fig. 1a). On blue light absorption, the modified Jα helix unfolds, thereby exposing the NES to nuclear CRM1 (exportin-1) receptors mediating active nuclear export through the nuclear pores.

In contrast to previous work on *As*LOV2-photocaged peptides^4^,^5^,^12^,^13^,^14^, we applied a new approach termed ‘Jα helix topping', which highly preserves wild-type *As*LOV2 Jα helix properties required for successful sequence epitope caging and *As*LOV2 domain photoswitching (see Supplementary Note for details). Using synthetic biology principles, we performed two cycles of rational design and subsequent selection, thereby gradually introducing specific, known NES-like features derived from literature knowledge^15^ into the wild-type *As*LOV2 Jα helix (Supplementary Fig. 1 and Supplementary Note). The resulting 33 *As*LOV2-NES hybrids were screened by qualitatively investigating the nucleocytoplasmic translocation of corresponding NLS-mCherry-*As*LOV2-NES fusions in human embryonic kidney (HEK 293T) cells on pulsatile blue light irradiation (Supplementary Fig. 2a,b). The N-terminal constitutive NLS is added to accumulate the fusion protein in the nucleus in the dark. Unless stated otherwise, nucleocytoplasmic translocation was analysed using epifluorescence microscopy. To our surprise, introducing two single-point mutations (A542L and A549L) plus adding a C-terminal aspartic acid residue already converted the wild-type Jα helix into a weak photocaged NES (NES 8; Supplementary Figs 1 and 2b), for which the functionality could be highly improved by introducing only two additional mutations (A543L and P547A) (NES 21; Supplementary Figs 1 and 2b). A control construct bearing the wild-type Jα helix did not show nuclear export on illumination (Supplementary Fig. 2b). Co-expression of a H2B-GFP chromatin marker confirmed that the NLS-mCherry-*As*LOV2-NES 21 fusion localizes to the nucleus and not to a different organelle in the dark and is strongly exported on illumination (Fig. 1b,c). ‘LEXY' refers to the *As*LOV2-NES 21 variant in all subsequent experiments.

We performed a complete induction–recovery cycle consisting of 3 min of mCherry imaging only followed by 15 min of pulsatile blue light irradiation and 20 min recovery in the dark in HEK 293T expressing NLS-mCherry-LEXY. The observed mCherry export on irradiation was very fast with half-times below 1 min (Fig. 1d and Supplementary Fig. 3). Stopping illumination led to full recovery of the initial nuclear mCherry fluorescence within about 15 min due to dark state recovery of the *As*LOV2 domain (Fig. 1d). We observed similar kinetics in human cervix carcinoma (HELA) and murine hepatoma (Hepa 1–6) cells (Supplementary Fig. 4). The combination of fast export on irradiation and full recovery in the dark enabled repeated cycles of activation and recovery without significantly compromising the performance of LEXY over time (Fig. 1e, Supplementary Fig. 5a,b and Supplementary Movies 1 and 2).

One particular advantage of optogenetics is the unmet spatiotemporal precision by which the light trigger can be applied. We irradiated single, selected cells with a 458-nm laser beam using a confocal laser scanning microscope. Induced cells showed a fivefold decrease in nuclear mCherry fluorescence while non-induced cells present in the same field of view remained unaffected (Fig. 1f,g and Supplementary Movie 3).

This fold change is higher than the one seen with epifluorescence microscopy (∼2.5-fold change in nuclear fluorescence; compare Fig. 1d,g). This is not surprising considering that, in epifluorescence microscopy, there is a non-negligible contribution of fluorescent signal in the nucleus from the surrounding cytoplasm. Therefore, confocal microscopy can better reveal the dynamic range of LEXY.

To test the robustness of LEXY-mediated export when fused to different target proteins, we cloned two golden gate entry vectors pLEXY and pNLS-LEXY, both comprising a cytomegalovirus (CMV)-driven mCherry-LEXY expression cassette carrying a bacterial toxin (ccdB^16^) encoding gene at its 5′-terminus, flanked by BpiI (BbsI) sites (Supplementary Fig. 6a). pNLS-LEXY harbours a constitutive NLS preceding the *ccdB* gene, which is absent in pLEXY. Human codon-optimized sequences of a bacterial protein domain (LexA repressor DNA-binding domain), the P1 bacteriophage-derived Cre recombinase, as well as six different human proteins (Acp1, Sox2, Nxt1, Nanog, Cox17 and p21) were cloned into both entry vectors via BpiI, thereby replacing the ccdB death gene. Note that all sequences encoded wild-type polypeptides, that is, we preserved endogenous regulatory elements including NLS/NES sequences or protein–DNA-binding interfaces. We found at least one LEXY-tagged version for each protein that showed significant nuclear export on blue light induction (Supplementary Fig. 6b,c). LEXY was able to outcompete endogenous NLSs, which is reflected by the efficient light-dependent export observed for the transcription factors Sox2 and Nanog. We also found that the nuclear export kinetics is influenced by both the total protein size and its nature. This is exemplified by the relatively slow export kinetics of the mCherry-LEXY-tagged Cre recombinase, which has not only about twice the size (∼85 kDa) of NLS-mCherry-LEXY alone (∼45 kDa) but also binds to DNA in the nucleus, thus preventing faster export rates (Supplementary Fig. 7).

LEXY-mediated control of protein export can be easily combined with our previously reported LINuS method for optogenetic control of nuclear import^4^. When co-expressing NLS-mCherry-LEXY and NES-EGFP-LINuS in HEK 293T, we observed a complete inversion of the nucleocytoplasmic localization of the two fluorophores on blue light irradiation (Supplementary Fig. 8a–c and Supplementary Movie 4).

### Light-dependent inhibition of endogenous nuclear export

**Figure 2:**
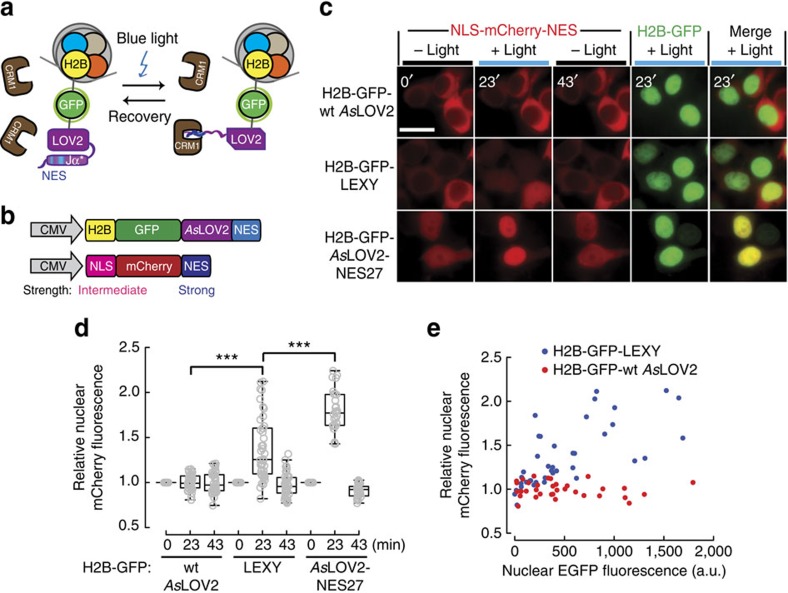
Light-dependent inhibition of endogenous nuclear export. (**a**) Schematic of blue light-dependent CRM1 sequestration by LEXY fused to H2B-GFP. (**b**) Schematic of constructs encoding H2B-GFP fused to LEXY and a NLS-mCherry-NES reporter. (**c**) Fluorescence images and (**d**) corresponding quantification of the relative nuclear mCherry fluorescence of cells co-transfected with the indicated constructs. Cells were incubated in the dark for 3 min followed by blue light irradiation for 20 min and dark recovery for 20 min. *As*LOV2-NES 27, a particularly strong NES variant (Supplementary Fig. 2b); wt, wild type. Data represent box plots and individual data points, *n*=34 cells for H2B-GFP-*As*LOV2, *n*=42 cells for H2B-GFP-LEXY and *n*=28 cells for H2B-GFP-*As*LOV2-NES27, all from 3 independent experiments; ***P=9.10 × 10^−10^ (wt *As*LOV2 versus LEXY) and 3.45 × 10^−5^ (LEXY versus *As*LOV2-NES27) by Mann–Whitney test. (**c**) Scale bar, 20 μm. (**e**) Scatter plot of the relative nuclear mCherry fluorescence versus nuclear EGFP fluorescence for the indicated samples in **d** after blue light irradiation (23 min time point).

Apart from direct light control of tagged proteins, LEXY could also be employed to perturb endogenous CRM1-dependent nuclear export. Anchoring LEXY to the nuclear chromatin by fusion to the histone H2B should enable light-dependent sequestration of endogenous CRM1 receptors (Fig. 2a). This should lead, in turn, to the inhibition of the nuclear export of CRM1 cargos. To verify this hypothesis we expressed H2B-GFP-LEXY alongside with a mCherry bearing a strong, constitutive NES and a weaker NLS in HEK 293T (Fig. 2b). We found that mCherry accumulated in the nucleus only in irradiated H2B-GFP-LEXY-expressing cells, but not in control cells expressing H2B-GFP fused to the wild-type *As*LOV2 domain (Fig. 2c,d). The extent of nuclear mCherry translocation depended on the abundance of H2B-GFP-LEXY (Fig. 2e). Using H2B-GFP fused to *As*LOV2-NES27, a particularly strong NES variant from our initial library (Supplementary Figs 1 and 2b), enhanced nuclear mCherry accumulation on irradiation, but also resulted in considerable leakiness, that is, an increased nuclear mCherry abundance in the dark (Fig. 2c,d). Overall, this strategy represents a genetically encoded, fully reversible alternative to chemical methods, such as, for example, leptomycin B (LMB) treatment^17^,^18^,^19^,^20^, widely employed in cell biology to block nuclear protein export.

### Regulation of synthetic transcriptional repressors

**Figure 3:**
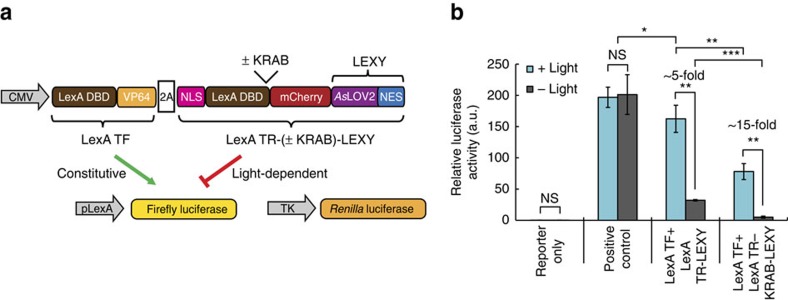
Light regulation of synthetic transcriptional repressors. (**a**) Schematic of used constructs for light control of transcriptional repression. DBD, DNA binding domain; KRAB, Krüppel associated box; LexA TF, synthetic LexA transcription factor; LexA TR-LEXY, LexA repressor fused to mCherry-LEXY; NLS, cMyc^P1A^ NLS; VP64, a strong transactivation domain; 2A, T2A peptide. (**b**) Quantification of relative luciferase activity in HEK 293T cells transiently transfected with the constructs in **a** and irradiated with blue light pulses for 24 h or incubated in the dark. Positive control, cells co-expressing LexA TF and NLS-mCherry-LEXY. Mean±s.d., *n*=4 independent experiments; *P<0.05, **P<0.01, ***P<0.001 by Welch's *t*-test. NS, not significant.

An interesting application of LEXY is the spatiotemporal control of transcriptional repressors by sequestering them into the cytosol, which could be of particular value for controlling endogenous gene activity. As proof of principle, we used the mCherry-LEXY-tagged LexA DNA-binding domain (Supplementary Fig. 6c) as passive mammalian repressor to control transgene expression with light. We also tested an additional LexA repressor variant carrying a Krüppel-associated box (KRAB), an active transcription repression domain^21^. A triple transfection into HEK 293T was performed with (1) a construct co-expressing the LEXY-tagged LexA repressor and a synthetic LexA-transcription factor required for reporter activation, (2) a corresponding LexA-dependent firefly luciferase reporter and (3) a constitutive *renilla* luciferase expression vector for normalization purposes (Fig. 3a). Following 24 h of pulsatile blue light irradiation we observed up to 15-fold increase in firefly luciferase expression as compared with the dark control, thereby confirming successful light regulation of the LexA repressors (Fig. 3b). Addition of the KRAB domain strongly reduced the background reporter activity in the dark, but also significantly compromised the total level of reporter activation on light induction (Fig. 3b).

### Optical control of p53 transcriptional activity

**Figure 4:**
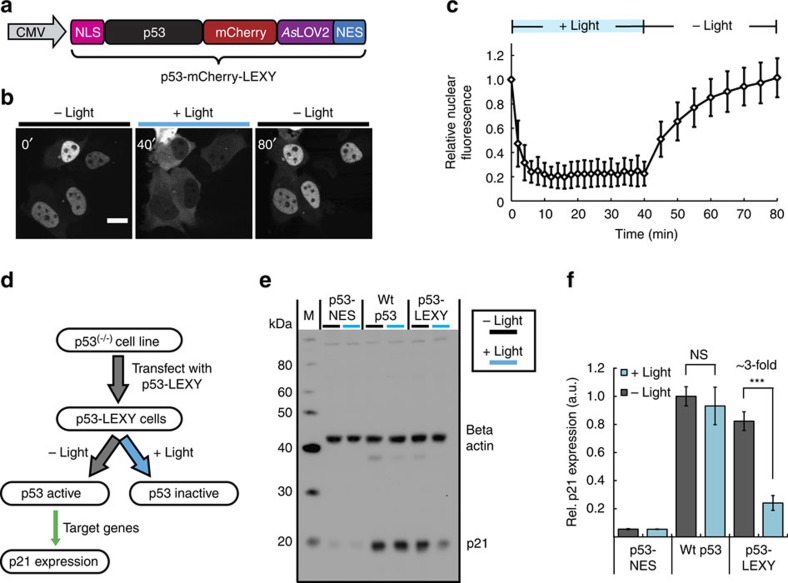
Optical control of p53 transcriptional activity. (**a**) Schematic of used vector for light control of human p53. NLS, cMyc^P1A^ NLS. (**b**) Fluorescence images and (**c**) corresponding quantification of the relative nuclear fluorescence over time of H1299 p53^−/−^ cells transfected with the construct in **a**. Cells were irradiated with blue light pulses for 40 min following a 40-min dark-recovery phase (mean±s.d., *n*=20 cells). (**b**) Scale bar, 20 μm. (**d**) Flowchart showing blue light control of p53 transcriptional activity. (**e**) Western blot analysis of p21 and beta-actin levels and (**f**) corresponding quantification for H1299 p53^−/−^ cells transiently transfected with p53-mCherry-LEXY or indicated control vectors and illuminated with blue light pulses for 48 h or incubated in the dark. NS, not significant; p53-LEXY, p53-mCherry-LEXY; p53-NES, p53-mCherry carrying a constitutive PKIt NES; wt p53, wild-type p53 fused to mCherry. (**f**) Mean±s.d., *n*=3 independent experiments; ***P=3.92 × 10^−4^ by Welch's *t*-test.

Finally, we sought to employ LEXY for direct optogenetic control of human p53, an important tumour suppressor frequently mutated in human cancers. It has been recently proposed that p53 dynamics control cellular signalling and therefore cell fate^22^. However, due to a lack of tools to spatiotemporally control p53 activity, rather indirect experimental strategies had to be employed to provoke specific dynamics of p53 activation, namely a combination of γ-irradiation and small-molecule (Nutlin) treatment^22^. Of note, wild-type p53 harbours several NLS and NES sequences and employs an NES masking/unmasking mechanism via tetramerization to regulate its nucleocytoplasmic localization^23^. The ubiquitin ligase Mdm2 furthermore regulates the overall p53 abundance in a close feedback manner^24^. Irrespective of its complex regulation, ectopic expression of p53 results in strong activation of p53 target genes^25^,^26^,^27^,^28^. Thus, we hypothesized that ectopic expression of LEXY-tagged p53 would enable direct optogenetic control of p53 activity. We fused p53 bearing an additional, N-terminal NLS to mCherry-LEXY and expressed the construct in human non-small cell lung carcinoma (H1299) cells harbouring a homozygous p53 deletion (p53^−/−^; Fig. 4a). Using confocal laser scanning microscopy we observed efficient and fully reversible blue light-induced nuclear export of the p53-mCherry-LEXY fusion (Fig. 4b,c). Remarkably, the dynamic range was comparable (∼5-fold change in nuclear fluorescence) to what we observed before for the NLS-mCherry-LEXY construct in HEK 293T (compare Fig. 4c and Fig. 1g). We then investigated the effect of light-mediated p53 translocation on the activation of p21, a prominent p53 target gene^25^,^26^. On treatment of p53-mCherry-LEXY-expressing H1299 cells with pulsatile blue light for 48 h (Fig. 4d) we observed a threefold decrease of p21 expression compared with the dark control, thereby confirming successful light regulation of p53 transcriptional activity (Fig. 4e,f). Control samples expressing wild-type p53 or p53 fused to a constitutive, strong NES did not show light-dependent p21 expression (Fig. 4e,f).

## Discussion

Here we reported the development of and showcased a variety of applications for LEXY, a light-inducible nuclear export system.

In contrast to previous studies^4^,^5^,^12^,^13^,^14^, we employed a novel approach termed ‘Jα helix topping' for photocaging a sequence epitope (here a NES) within the *As*LOV2 domain (see Supplementary Note for details). Overall, about ∼50% of all *As*LOV2-NES hybrids generated showed appreciable, light-dependent nuclear export (Supplementary Fig. 2b), indicating a high success rate of this approach. We speculate that ‘Jα helix topping' might prove useful also for *As*LOV2 photocaging of other peptides, although this will surely depend on the nature of the peptides to be photocaged and the knowledge available on their design.

Remarkably, the so-obtained light-inducible nuclear export signal—LEXY—mediated robust, fully reversible blue light-dependent nuclear export in all cell lines and in combination with all soluble proteins tested so far. The fivefold change in nuclear mCherry fluorescence on blue light induction observed when using confocal microscopy (Fig. 1g) indicates a high dynamic range of the system, especially considering that this construct is still prone to passive diffusion due to its relatively small size (45 kDa), which is below the passive diffusion limit of the nuclear pores^29^. Apart from selecting *As*LOV2-NES hybrids matching the users' needs from our library (Supplementary Figs 1 and 2b), introducing well-known mutations that enhance Jα helix stability and photocaging^12^,^13^ might enable toggling LEXYs' dynamic range and photocaging properties further, in case this were required for a specific application.

When fused to target proteins, LEXY mediated nuclear export, regardless of endogenous regulation present (for example, NLSs/NESs or DNA-/protein-binding sites), indicating tight caging and high strength of the engineered, photocaged NES. This favourable property enabled straightforward control of nuclear export of different wild-type human proteins, including the widely studied and frequently mutated tumour suppressor p53, but also other transcription factors (Sox2 and Nanog), a phosphatase (Acp1), a protein export-related factor (Nxt1), a cyclin-dependent kinase inhibitor (p21), a copper chaperone (Cox17), as well as a bacterial repressor (LexA) and a bacteriophage-derived protein (Cre recombinase). Given the high degree of conservation of the nuclear import and export machineries amongst eukaryotes^30^,^31^, we believe that LEXY will be applicable in various cellular contexts and organisms to control nuclear export of in principle any protein of interest, regardless of its inherent nature, size or endogenous regulation of its nucleocytoplasmic trafficking.

An important, shared feature of LEXY and the previously reported tools for optogenetic control of nuclear import, LINuS^4^ and LANS^5^, is their minimalistic, *As*LOV2-based, single-component design, which highly simplifies their application for light control of fully functional, wild-type proteins. This represents an important advantage as compared with most optogenetic dimerizers that typically require splitting functional proteins into parts before reconstituting them with light^32^,^33^,^34^. Furthermore, the single-component nature of LEXY and LINuS allowed us to apply both in the same cell, so that we could simultaneously import and export two fluorescent proteins with the same blue light trigger (Supplementary Fig. 8). This example is the first conceptual step towards parallelized optogenetic control of multiple proteins acting at opposing cellular locations.

Recently, it has been shown that a single-component optogenetic tag for light-inducible nuclear shuttling (LANS) could be genomically integrated using CRISPR/Cas genome editing technology^35^,^36^,^37^ to control the import of an endogenous transcription factor in *Caenorhabditis elegans*^5^. From the pure structural perspective, LEXY and LANS (as well as our previously published LINuS tag^4^) only differ in the type of peptide photocaged within the *As*LOV2 domain. Thus, we speculate that LEXY might be equally well suited for direct genomic tagging and thereby enable light control of endogenous proteins.

Since decades, LMB, a fungus-derived antibiotic^19^, is widely used to block CRM1-dependent export in eukaryotes^18^,^20^—for instance, to investigate if a protein of interest is shuttling between the nucleus and the cytoplasm^38^,^39^,^40^,^41^,^42^,^43^. More recently, LMB and analogues gained attention as potential anticancer agents^44^,^45^,^46^, likely functioning by reactivating the p53 pathway in cancers with increased p53 export rates^47^,^48^,^49^. Unfortunately, LMB is toxic due to its direct, irreversible binding to CRM1 receptors^17^,^20^,^50^, thereby compromising its application in animal models and human patients. Furthermore, being a small chemical inhibitor, it cannot be used for dynamic, spatiotemporal control of nuclear export.

By using chromatin-anchored LEXY variants (H2B-GFP-LEXY and H2B-GFP-*As*LOV2-NES27), we could inhibit nuclear export of a CRM1-dependent cargo (a shuttling mCherry; Fig. 2c,d). This method can be, therefore, used to perturb the nucleocytoplasmic distribution of endogenous proteins and could be of great value for instance to dissect the role of altered nuclear export rates during malignant transformation in space and time. Importantly, the ‘opto-LMB' presented here has the great advantage of being tunable by selecting NESs of a specific strength to be photocaged in the *As*LOV2 domain, so that only cargos with NESs of comparable or weaker strengths are affected.

A similar approach has been previously used to recruit mCherry to chromatin by triggering the interaction between chromatin-anchored UVR8 and NLS-mCherry-COP1 with ultraviolet light^3^. In this study, however, we recruit to chromatin endogenous proteins—the CRM1 receptors—thereby employing the photocaged NES as inducible and specific ‘protein fishing rod'. It would be of great interest to further investigate this concept also in the context of other endogenous proteins—for instance, chromatin modifiers. In theory, fusing *As*LOV2-photocaged peptides binding such proteins to Cas9 might enable their highly dynamic light recruitment to specific genomic loci. However, this would require engineering corresponding *As*LOV2-photocaged peptides that bind the endogenous proteins to be recruited with high affinity and specificity.

In sum, LEXY is an important addition to the optogenetic toolbox for interrogating the spatiotemporal dynamics of cellular signalling^51^. We believe that LEXY will provide the wide cell biology community with robust light control of many different proteins of interest and will enable novel studies on how nucleocytoplasmic protein dynamics impact cellular responses.

## Methods

### Plasmid construction

Constructs were generated using classical restriction enzyme cloning or golden gate cloning. Oligonucleotides were obtained from Sigma-Aldrich and codon-optimized DNA sequences were obtained as gBlocks from Integrated DNA Technologies. Golden gate cloning has been described previously by others^52^. A list of constructs used in this study (Supplementary Table 1), oligonucleotide sequences (Supplementary Table 2) and protein-encoding sequences created in this study (Supplementary Data) are provided as Supplementary Information. Constructs are available via Addgene (plasmids #72655–72662) or on request.

For screening of the different *As*LOV2-NES hybrid variants, vectors expressing NLS-mCherry-*As*LOV2-NES fusions were cloned. As template, we used pDN34 (previously reported by us^4^) encoding a mCherry-*As*LOV2 fusion harbouring a PKIt NES N-terminally, and an artificial, bipartite NLS (biNLS2; ref. 4) photocaged within the *As*LOV2 Jα helix C-terminally. First, we replaced the N-terminal NES with a cMyc^P1A^ NLS (AAAKRVKLD). Therefore, the complete pDN34 vector (excluding the PKIt NES) was PCR amplified using oligos 1 and 2 following digestion of the amplicon with BsmBI. The cMyc^P1A^ NLS was then introduced by oligo cloning using oligos 3 and 4, thereby yielding vector pDN101. The biNLS2 in pDN101 was then replaced by different artificial Jα-NES hybrid sequences (Supplementary Fig. 1). Therefore, the whole vector pDN101 excluding the biNLS2 sequence was PCR amplified with oligos 5 and 6 following digestion of the amplicon with BsmBI. Then NES 1–33 were introduced by oligo cloning using oligos 7–72 pairwise, thereby generating vectors pDN102–pDN134 encoding 33 different NLS-mCherry-*As*LOV2-NES fusion variants. A control construct bearing the wild-type *As*LOV2 domain instead of a *As*LOV2-NES hybrid was generated accordingly using oligo pair 73–74, thereby yielding construct pDN135.

Next, we generated a vector co-expressing H2B-GFP and NLS-mCherry-LEXY (harbouring the *As*LOV2-NES21 hybrid) using a 2A peptide co-expression strategy^53^,^54^,^55^. Therefore, we PCR amplified the whole vector pDN122 using oligos 2 and 75. A fragment encoding H2B-GFP was PCR amplified from vector H2B-GFP^56^ (kind gift from Geoff Wahl (Addgene plasmid #11680)) using oligos 76/77. Note that oligos 75 and 77 encode complementing halves of a T2A peptide^54^ sequence. The resulting amplicons were digested with BsmBI and ligated, thereby generating construct pDN136.

To simplify cloning of mCherry-LEXY-tagged proteins of interest, we generated two golden gate entry vectors, namely pLEXY (pDN137) and pNLS-LEXY (pDN138). These vectors encode a CMV promoter-driven mCherry-LEXY expression cassette preceded by a bacterial toxin-encoding gene (*ccdB*^16^). The ccdB is flanked by BpiI (BbsI) sites and can thus be easily replaced by protein-encoding sequences using golden gate cloning. pNLS-LEXY furthermore contains a cMyc^P1A^ NLS 5′ of the *ccdB* gene, which is absent in pLEXY. We amplified a BpiI-flanked ccdB-encoding sequence from pDonor (Invitrogen) using oligos 78 and 80. We also amplified BpiI-flanked ccdB preceded by a cMyc^P1A^ NLS using oligos 79 and 80. mCherry-LEXY was PCR amplified from pDN122 in two fragments using oligos 81/82 and 83/84. Note that primers lead to mutagenesis and thereby removal of the BpiI site present within the mCherry-coding sequence. All amplicons were digested with BsmBI. Vector H2B-GFP^56^ was digested with EcoRI/NotI, thereby removing the H2B-GFP insert. Ligation of both mCherry-LEXY fragments with either ccdB fragment (with or without 5′ cMyc^P1A^ NLS) was performed into the EcoRI/NotI-linearized H2B-GFP vector, thereby yielding vectors pDN137 and pDN138. Note that BpiI digestion of pDN137 (pLEXY) and pDN138 (pNLS-LEXY) yields the following, identical overhangs: 5′: CATG; 3′: GTGA. When introducing a protein of interest by golden gate cloning, the 5′ CATG overhang corresponds to the start codon (underlined). The additional 5′ cytosine is either part of the Kozak consensus sequence (in pLEXY) or represents the last base of the last cMyc^P1A^ NLS codon (GAC encoding the aspartic acid residue; in pNLS-LEXY).

To generate mCherry-LEXY-tagged proteins, human codon-optimized sequences encoding p21 (Uniprot ID #P38936), Sox2 (#P48431), Nanog (#Q9H9S0), Cox17 (#Q14061), Acp1 (#P24666), Nxt1 (#Q9UKK6), Cre recombinase (#P06956) and the LexA DNA-binding domain (LexA residues 1–87; #P0A7C2) were ordered as gBlocks from Integrated DNA Technologies. All gBlocks were flanked by BpiI sites yielding 5′ CATG and 3′ GTGA overhangs. Each gBlock was then introduced by golden gate cloning into pNLS-LEXY and pLEXY via BpiI, thereby replacing the *ccdB* gene and generating vectors pDN139–pDN154.

To test the compatibility of LEXY and LINuS^4^-mediated protein translocation, a vector co-expressing NES-EGFP-LINuS and NLS-mCherry-LEXY was constructed. We first generated a NES-EGFP-LINuS template vector by PCR amplifying the enhanced green fluorescent protein (EGFP) insert from pEGFP-N1 (Clontech) using oligos 85 and 86. The resulting amplicon was digested with NheI/BsrGI and ligated into a NheI/BsrGI-linearized pDN77 (previously reported by us^4^), yielding vector pDN155. Subsequently, a fragment comprising cMyc^P1A^-NLS-mCherry-*As*LOV2-NES21 was PCR amplified from pDN122 using oligos 75 and 87. Vector pDN155 was PCR amplified completely using oligos 88 and 89. Note that oligos 75 and 88 encode complementing halves of a T2A peptide^54^ sequence. Amplicons were digested with BsmBI and ligated, thereby generating vector pDN156 encoding IkBα_NES-EGFP-biLINuS2-T2A-cMyc^P1A^_NLS-mCherry-*As*LOV2-NES21.

For blue light-dependent sequestration of endogenous CRM1 receptors, fusions of *As*LOV2-NES hybrids or a wild-type *As*LOV2 domain (as control) to H2B-GFP were created. Therefore, vector H2B-GFP was first digested with BsrGI/NotI, thereby linearizing the vector directly 5′ of the H2B-GFP stop codon. Vectors pDN122, pDN128 and pDN135 were digested with BsrGI/NotI and the *As*LOV2-NES or wild-type *As*LOV2-encoding fragments were purified. Subsequently, each *As*LOV2-NES or wild-type *As*LOV2-encoding fragment was ligated into the linearized H2B-GFP vector. The resulting vectors pDN157–pDN159 encode H2B-GFP N-terminally fused to the *As*LOV2-NES21 hybrid, *As*LOV2-NES27 hybrid or the wild-type *As*LOV2, respectively.

A NLS-mCherry-NES reporter was generated by PCR amplifying the whole NLS-mCherry-*As*LOV2-NES-encoding vector pDN118 with oligos 90 and 91, thereby removing the *As*LOV2 domain. The resulting amplicon was digested with BsmBI and religated, yielding a NLS-mCherry-NES-encoding vector bearing a cMyc^P1A^ NLS N-terminally and a strong, constitutive NES C-terminally (pDN160).

For constructing of LEXY-tagged LexA repressors, we first generated a golden gate entry vector consisting of a CMV promoter followed by a *ccdB* gene flanked with BpiI sites. Therefore, a fragment comprising the ccdB toxin-encoding gene was PCR amplified from vector pDN137 using oligos 92/93. The amplicon was digested with EcoRI/NotI and ligated into an EcoRI/NotI-linearized vector H2B-GFP, thereby yielding pDN161 (pANY-entry). Note that BpiI digestion of pANY-entry generates 5′ CATG (with ATG (underlined) representing the start codon) and 3′ TGAC (with TGA (underlined) representing the stop codon) overhangs.

Subsequently, constructs co-expressing a synthetic LexA-transcription factor (that is, the LexA DNA-binding domain fused to a strong VP64 transactivator) and either NLS-mCherry-LEXY (as control) or a NLS-LexA-mCherry-LEXY repressor with or without an additional KRAB domain (that is, amino acids 1–75 from human zinc finger protein 10; Uniprot ID #P21506) were constructed. Therefore, human codon-optimized sequences encoding LexA-VP64-T2A (g1) and NLS-LexA-mCherry-LEXY (g2), NLS-LexA-KRAB-mCherry-LEXY (g3) or NLS-mCherry-LEXY (g4) fusions were ordered as gBlocks from Integrated DNA Technologies. All sequences were flanked by BpiI sites. Subsequently, gBlocks were assembled pairwise (g1–g2, g1–g3 and g1–g4) into pDN161 using golden gate cloning, thereby generating vectors pDN162–pDN164. A corresponding LexA-dependent firefly luciferase reporter (pDN100) was reported earlier by us^4^. A constitutive *renilla* expression vector (pRL-TK) was obtained from Promega.

For tagging p53 with mCherry-LEXY, a human p53-encoding sequence (Uniprot ID #P04637) was PCR amplified from vector p53 using oligos 94 and 95. The resulting amplicon was digested with BsmBI and ligated into a BpiI-linearized vector pDN138 (pNLS-LEXY), thereby yielding vector pPW1. Note that the resulting NLS-p53-mCherry-LEXY fusion harbours a constitutive N-terminal cMyc^P1A^ NLS.

Subsequently, control vectors expressing p53-mCherry (pPW2) or p53-NES-mCherry bearing a constitutive PKIt NES (pPW3) were generated. Therefore, a p53-encoding sequence was PCR amplified from vector p53 with oligos 96/97 following digestion of the amplicon with HindIII/BamHI. A fragment encoding mCherry or NES-mCherry was amplified from pmCherry-N1 (Clontech) using oligos 98/100 and 99/100, respectively, following digestion with BamHI/XhoI. The p53 fragment was ligated with either mCherry fragment (with or without NES) into a HindIII/XhoI-linearized pcDNA3.1 (+) vector (Invitrogen), thereby generating constructs pPW2 and pPW3.

### Cell culture and transient transfection

Human embryonic kidney (HEK 293T; kindly provided by Dirk Grimm, Heidelberg University Clinics), human cervix carcinoma (HELA; kindly provided by Kathleen Börner, Heidelberg University Clinics) and murine hepatoma (Hepa 1–6; kindly provided by Stephan Herzig, Helmholtz Diabetes Center, Munich) cells were maintained in phenol red-free Dulbecco's Modified Eagle Medium supplemented with 10% fetal calf serum (Biochrom AG, Berlin, Germany), 2 mM L-glutamine (Invitrogen/Gibco), 100 U ml^−1^ penicillin and 100 μg ml^−1^ streptomycin (Invitrogen/Gibco). HEK 293T and HELA cells were selected for LEXY characterization experiments as they can be efficiently transformed even with very gentle transfection reagents, thereby avoiding unnecessary cellular stress or toxicity that could affect experimental outcomes. Human non-small cell lung carcinoma cells harbouring a homozygous partial p53 deletion (H1299; kindly provided by Alexander Loewer, Max Delbrück Center for Molecular Medicine, Berlin) were maintained in RPMI 1640 media (Sigma-Aldrich) supplemented with 10% fetal calf serum, 100 U ml^−1^ penicillin and 100 μg ml^−1^ streptomycin. Cells were cultivated at 37 °C and 5% CO_2_ and were passaged when reaching ∼90% confluency. Before usage, all cell lines were authenticated and tested for mycoplasma contamination using the commercial Multiplex Cell Line Authentication and Mycoplasma Test services (Multiplexion, Heidelberg, Germany; http://www.multiplexion.de/en/). Mycoplasma contamination testing was repeated yearly using a PCR Mycoplasma Test Kit (PromoKine).

For microscopy experiments in HEK 293T, HELA and Hepa 1–6, cells were seeded into 35-mm glass-bottom dishes (Greiner BIO-ONE) at densities of ∼150,000 (for HELA), ∼300,000 (for HEK 293T) and ∼500,000 (for Hepa 1–6) cells per dish. The following day, cells were transfected with 500 ng (for HEK 293T or HELA) or 2,000 ng (for Hepa 1–6) total DNA using JetPrime (Polyplus-transfection) according to the manufacturer's instructions. For shuttling experiments of different LEXY fusions (including the initial screen of NLS-mCherry-*As*LOV2-NES variants), the LEXY fusion-encoding vector and a pBluescript II SK stuffer were co-transfected in a ratio of 1:9. In some experiments, an H2B-GFP-expressing vector was additionally co-transfected in a ratio LEXY fusion:H2B-GFP:stuffer of 1:1:8. Microscopy analysis was performed 24 h post transfection. For inhibition of nuclear export by light sequestration of endogenous CRM1, H2B-GFP-LEXY and H2B-GFP-*As*LOV2-NES27 vectors or an H2B-GFP-wild-type *As*LOV2 control vector were co-transfected with the NLS-mCherry-NES reporter in a ratio of 9:1 and microscopy started 48 h post transfection to ensure high expression of the H2B fusion. For light control of p53 translocation, H1299 p53^−/−^ cells were seeded into 35-mm glass-bottom dishes at a density of 200,000 cells per dish. The next day, co-transfection was performed with 100 ng of p53-mCherry-LEXY expression vector and 900 ng of pBluescript II SK stuffer using Lipofectamine 2000 (Invitrogen) according to the manufacturer's recommendations. Microscopy started 24 h post transfection. Note that post transfection and before microscopy all samples were incubated and transported in light shielding chambers to avoid premature activation due to white light exposure. Transfections for light control of LexA repressor-dependent transgene expression and p53 transcriptional activity are described in the corresponding paragraphs below.

### Epifluorescence microscopy

Epifluorescence microscopy was performed using a DeltaVision system (Applied Precision) comprising an Olympus IX inverted microscope (Olympus) equipped with a HBO 100 W mercury arc lamp light source (Olympus) and a CoolSnap HQ charge-coupled device camera (Photometric). Images were acquired at 5% CO_2_ and 37 °C using a × 63/1.40 numerical aperture oil objective (Olympus). For blue light irradiation as well as imaging of EGFP, the FITC channel (wavelengths/bandwidths (in nm): excitation 490/20, emission 528/38) was used. mCherry was imaged using the RD-TR-PE filter set-up (excitation 555/28, emission 617/73).

Cells were focused in the mCherry channel, thereby avoiding white light irradiation before imaging. Time-lapse microscopy was performed applying automated imaging time courses, typically comprising a 3-min pre-induction phase, a 15–20-min blue light induction phase and an optional 20-min dark-recovery phase. During pre-induction or dark-recovery phase, only mCherry images were taken every 30 s. For blue light irradiation, 1-s blue light pulses were applied every 30 s by taking an image in the FITC channel (using 1 s exposure time and 100% light intensity), preceded by taking an mCherry image before each blue light pulse. For imaging of EGFP and mCherry in parallel during the blue light induction phase (Figs 1c and 2c,e and Supplementary Fig. 8b,c), an mCherry image was taken every 30 s, each following two images in the FITC channel taken with 850–930 and 70–150 ms exposure times (the latter was used as image for EGFP data analysis). Note that the sum of the FITC channel exposure times representing the total blue light pulse length was always 1 s.

### Confocal laser scanning microscopy and single-cell activation

Activation of NLS-mCherry-LEXY translocation in single cells (Fig. 1f,g) was performed using a Leica Sp5 confocal microscope system equipped with automated temperature (37 °C) and CO_2_ (5%) control, a multiline argon laser and a PL Apo CS × 40 oil objective (numerical aperture=1.3). Cells were focused using the mCherry signal activated with the 561-nm laser line and a circular region of interest (ROI; ∼30 μm^2^) was placed onto single, selected cells. The ROI was scanned with a 458-nm laser beam (∼2 μW intensity) for 30 ms every 10 s for 10 min following a 20-min dark-recovery phase. mCherry was imaged in parallel every 10 s for 30 min using the 561 laser line for excitation. Laser intensity was measured with a Laser Power and Energy meter (Nova). p53-mCherry-LEXY translocation (Fig. 4b,c) was induced by irradiating a whole field of view with a 458 nm laser beam (intensity ∼2 μW) every 30 s for 40 min following a 40-min dark-recovery phase. In parallel, mCherry images were taken every 30 s during the blue light induction and every 5 min during the dark-recovery phase.

### Image data processing and analysis

Images were processed in ImageJ (version 1.46r; http://imagej.nih.gov/ij/) first by bleach-correcting the raw data using the Bleach Corrector plugin (http://cmci.embl.de/downloads/bleach_corrector; developed by Kota Miura and Jens Rietdorf, European Molecular Biology Laboratory, Heidelberg). Subsequently, nuclei were segmented manually by drawing a circular ROI or automatically. For automated segmentation, an automated threshold was applied to locate nuclei in the EGFP channel (H2B-EGFP), which were subsequently tracked over time using the ImageJ particle analyser. Locations of nuclei were then used to measure the nuclear mCherry fluorescence intensity for each cell at each time point. The relative nuclear fluorescence was calculated by normalizing the resulting mCherry fluorescence intensity values to the initial intensity values at time point 0 (that is, beginning of experiment) for each cell.

### Light control of artificial repressors and luciferase assay

HEK 293T cells were seeded into black, clear bottom 96-well plates (Corning) at a density of ∼12,000 cells per well. Cells were co-transfected with 10 ng of a construct co-expressing a constitutive LexA-transcription factor and a mCherry-LEXY-tagged LexA repressor (or mCherry-LEXY as control), 10 ng of LexA-dependent firefly luciferase reporter (pDN100, reported previously^4^), 0.1 ng of a constitutive *renilla* expression construct (pRL-TK; Promega) and 30 ng of stuffer DNA (pBluescript II SK) using JetPrime according to the manufacturer's instructions (DNA amounts are per well).

Fifteen hours post transfection, cells were incubated in the dark or illuminated for 24 h with 460 nm pulsatile blue light (5 s ON, 15 s OFF; light intensity: 20 μmol s^−1^ m^−2^ as measured with a LI-COR LI-250A Light Meter). A custom-made LED device composed of six high-power LEDs (type CREE XP-E D5–15; LED-TECH.DE) empowered by a Switching Mode Power Supply (Manson, model: HCS-3102) served as light source. The HCS software (Manson, version 0.9) was used for automated control of blue light intensity and generation of the pulsatile illumination regime. Subsequently, a dual luciferase assay was performed using the Dual-Glo luciferase assay kit (Promega) according to the manufacturer's protocol. In brief, cells were collected into the supplied lysis buffer, and firefly and *renilla* luciferase activities were quantified using a GLOMAX 96 Microplate Luminometer (Promega) with automated injectors (delay time was 2 s and integration time 10 s for firefly and *renilla*). The relative luciferase activity was calculated by normalizing firefly to *renilla* luciferase photon counts.

### Light control of p53 activity and western blot

H1299 p53^−/−^ cells were seeded into six-well plates at a density of ∼100,000 cells per well. The next day, cells were co-transfected with 100 ng of p53-mCherry-LEXY, p53-NES-mCherry or p53-mCherry expression vectors, respectively, and 900 ng of pBluescript II SK stuffer using Lipofectamine 2000 according to the manufacturer's recommendations. Following transfection, cells were irradiated with blue light pulses as described above (light intensity: 20 μmol s^−1^ m^−2^) for 48 h or incubated in the dark. Note that blue light irradiation started directly after transfection. Next, cells were collected into ice-cold lysis buffer (20 mM Tris-HCl, pH 7.4, 1% Triton X-100, 10% glycerol, 150 mM NaCl, 1% phenylmethylsulfonyl fluoride, 1% Benzonase (Novagen) and 1 Complete Mini Protease Inhibitor tablet (Roche)) followed by protein separation by SDS–PAGE. Proteins were then transferred onto a polyvinylidene difluoride membrane and the membrane was blocked using 5% milk in PBS-T. Primary antibodies were diluted in 5% milk in PBS-T and applied for 1 h to detect p21 (BD Pharmingen, #556430, diluted 1:666) and beta-actin (Abcam, #ab8226, diluted 1:1,000), followed by incubation with a secondary goat anti-mouse IgG (H+L)-PRPO (Dianova, 115-035-003) for 45 min. Chemiluminescence was detected using the SuperSignal West Pico Chemiluminescent Substrate (Thermo Scientific) and the ChemoCam Imager (Intas). The relative p21 expression was calculated by quantifying western blot band intensities using the ImageJ Gel Analyzer plugin. p21 levels were normalized to beta-actin levels for each sample followed by normalization to the wild-type p53 control in the dark.

### Statistical analysis

Independent replicates refer to independent cell samples seeded, transfected, treated and analysed on different days. Uncertainties in the reported mean values are indicated as the s.d. or s.e.m. as stated in the figure legends. The differences in reported values were tested for statistical significance using a (two-sided) Welch's unequal variances *t*-test (Welch's *t*-test). For not normally distributed data, a Mann–Whitney test was applied. *P* values <0.05, 0.01 and 0.001 were considered statistically significant and are indicated with 1, 2 and 3 asterisks, respectively. *P* values ≥0.05 were considered statistically not significant.

### Reproducibility

For each experiment, sample sizes were chosen based on an initial pilot experiment. Similar experiments reported in previous publications were further used to direct sample sizes. No data were excluded from the analysis. No blinding or randomization was used in the course of the experiments.

## Additional information

**How to cite this article:** Niopek, D. *et al*. Optogenetic control of nuclear protein export. *Nat. Commun.* 7:10624 doi: 10.1038/ncomms10624 (2016).

## Supplementary Material

Supplementary Figures, Supplementary Tables, Supplementary Note and Supplementary References Supplementary Figures 1-8, Supplementary Tables 1-2, Supplementary Note 1 and Supplementary References

Supplementary Data 1 Sequences of constructs used in this study

Supplementary Movie 1 Repeated activation of nuclear export in HEK 293T. Cells expressing NLS-mCherry-LEXY were repeatedly irradiated with blue light pulses (indicated by “+ light”) followed by recovery in the dark. Fluorescence images of mCherry were taken every 30 s. Numbers indicate hours:minutes:seconds.

Supplementary Movie 2 Repeated activation of nuclear export in HELA. Cells expressing NLS-mCherry-LEXY were repeatedly irradiated with blue light pulses (indicated by “+ light”) followed by recovery in the dark. Fluorescence images of mCherry were taken every 30 s. Numbers indicate hours:minutes:seconds.

Supplementary Movie 3 LEXY activation can be spatially confined. Selected HEK 293T cells expressing NLS-mCherry-LEXY were irradiated with 1 s blue light pulses every 10 s for 10 min by scanning the indicated region-of-interest (green circles) with a 458 nm laser beam followed by 20 min recovery in the dark. Fluorescence images of mCherry were captured every 10 s. Numbers indicate minutes:seconds.

Supplementary Movie 4 Parallelized optogenetic control of nuclear import and export. HEK 293T cells co-expressing NES-EGFP-LINuS and NLS-mCherry-LEXY were irradiated with 1 s blue light pulses every 30 s for 15 min. Fluorescence images of EGFP and mCherry were captured every 30 s. Numbers indicate minutes:seconds.
