# Supplementary material for: Optogenetic control of nuclear protein export
Source: Nat Commun. 2016 Feb 8;7:10624. doi: 10.1038/ncomms10624 (PMC4748110; doi:10.1038/ncomms10624)
Supplement: Supplementary Figures, Supplementary Tables, Supplementary Note and Supplementary References — Supplementary Figures 1-8, Supplementary Tables 1-2, Supplementary Note 1 and Supplementary References [file ncomms10624-s1.pdf]

Supplementary Figures

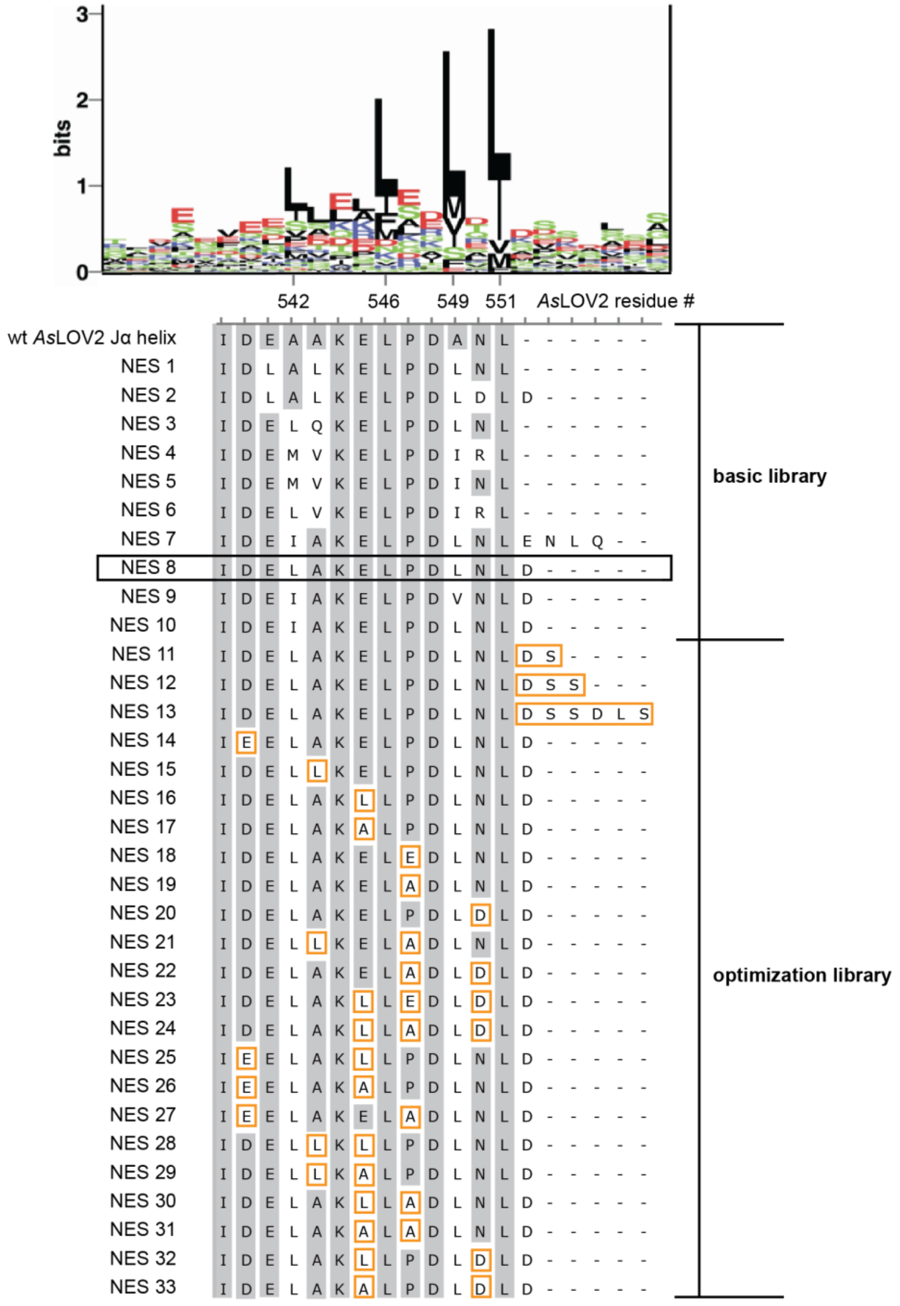

Supplementary Figure 1 | Library of artificial *As*LOV2-NES hybrids. Top: sequence logo derived from multiple sequence alignment of 58 experimentally

validated NESs (adapted from la Cour *et. al*, 2003)[1]. Below: alignment of artificial *As*LOV2-NES hybrids variants to the wild type (wt) *As*LOV2 Jα helix residues 539-551. The NES 8 variant (black rectangle) was used as lead candidate for generating the optimization library (NESs 11-33). Residues shaded in grey are identical to corresponding wt *As*LOV2 residues. Orange rectangles depict residues in NESs 11-33 that were altered as compared to the lead candidate NES 8.

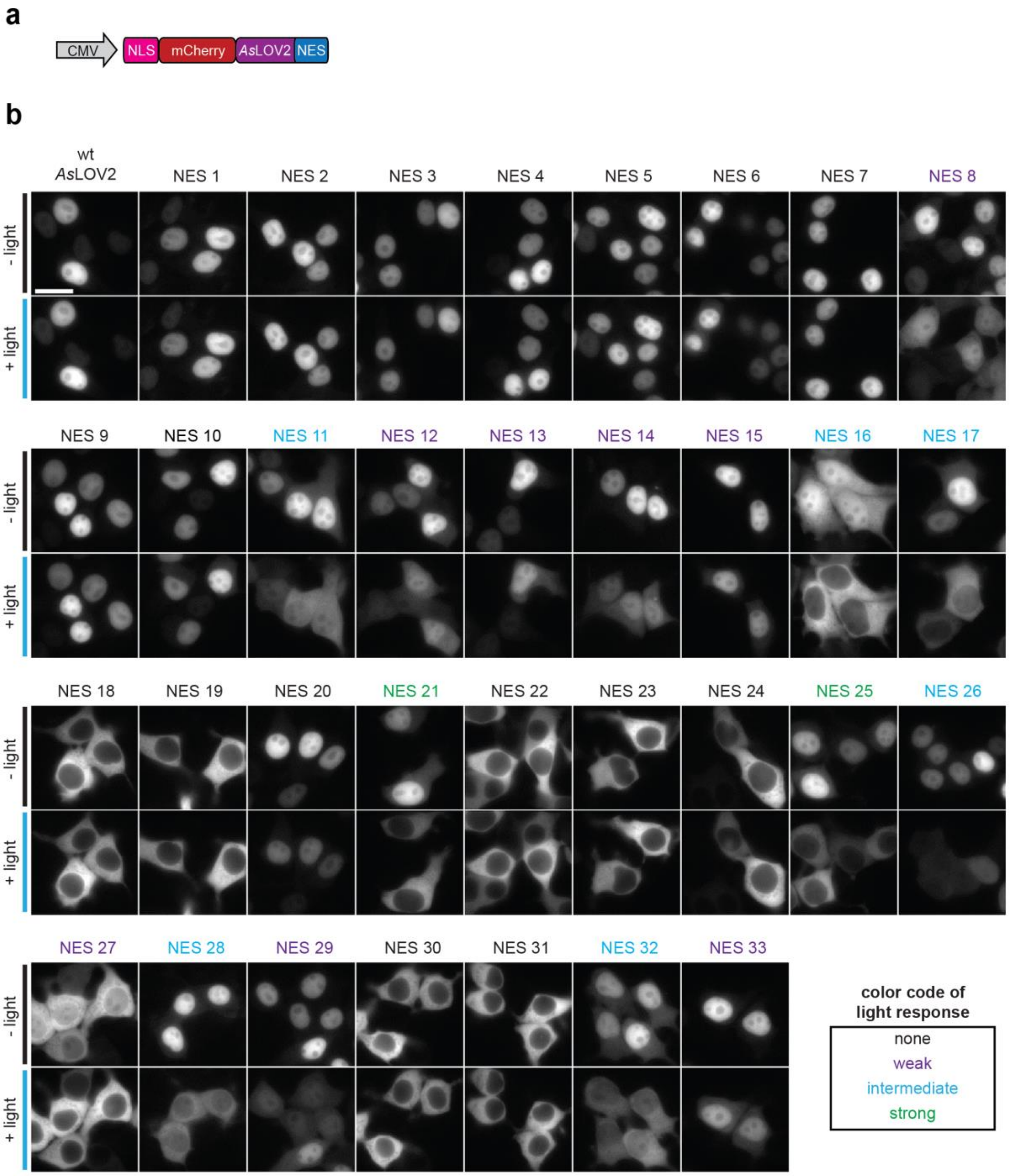

Supplementary Figure 2 | Qualitative screen of *As*LOV2-NES hybrids in HEK 293T cells. (**a**) Schematic of the general construct used. Each specific construct carries a distinct Jα helix/NES hybrid whose sequence is shown in **Supplementary Fig. 1**. NLS: cMyc^{P1A} NLS. CMV: immediate early cycomegalovirus promoter. (**b**)

Fluorescence microscopy images of cells transiently transfected with the indicated NLS-mCherry-*As*LOV2-NES variant or a corresponding control construct bearing the wild type *As*LOV2 domain (wt *As*LOV2) prior to induction (- light) and after 15 min of illumination with blue light pulses (+ light). Scale bar, 20 µm.

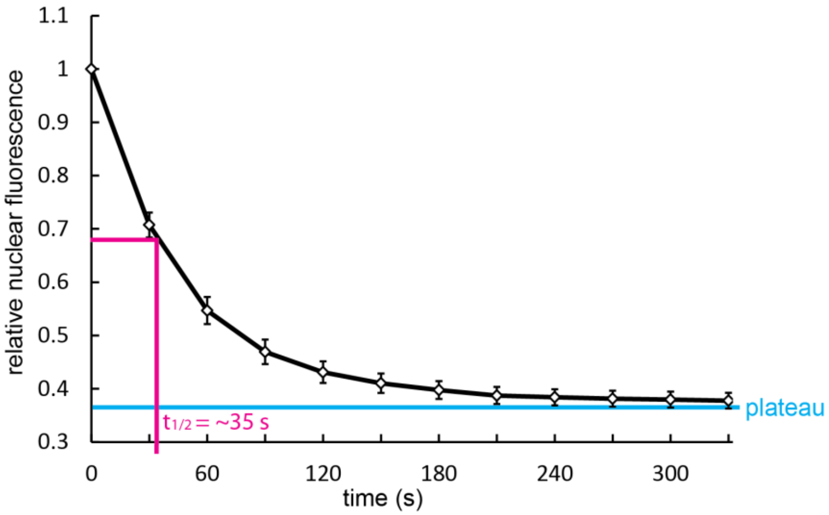

Supplementary Figure 3 | Estimation of NLS-mCherry-LEXY nuclear export half-time in HEK 293T. Cells expressing NLS-mCherry-LEXY were induced with blue light pulses for 15 min. Only the first 330 s after starting the light exposure are shown (mean ± s.e.m, n = 22 cells, 3 independent experiments).

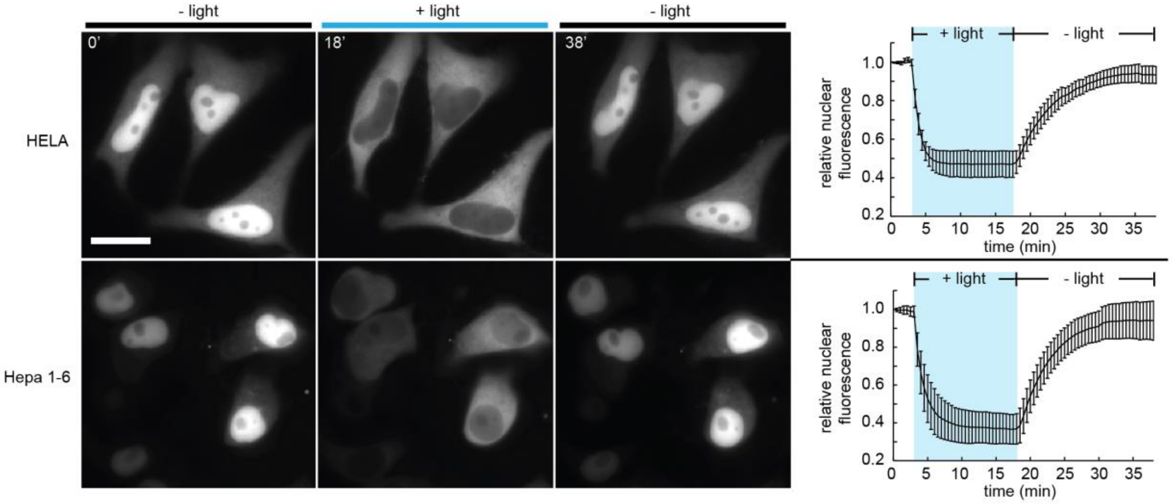

Supplementary Figure 4 | LEXY functions in different cell lines. HELA cells and murine Hepa 1-6 cells transiently transfected with NLS-mCherry-LEXY were incubated in the dark for 3 min, then induced with blue light for 15 followed by a 20 min recovery phase in the dark. Left: fluorescence microscopy images at the indicated time points. Scale bar, 20 µm. Right: Quantification of relative nuclear localization over time (mean ± SD, n = 14 cells, 2 independent experiments for HELA, and n= 24 cells, 3 independent experiments for Hepa 1-6).

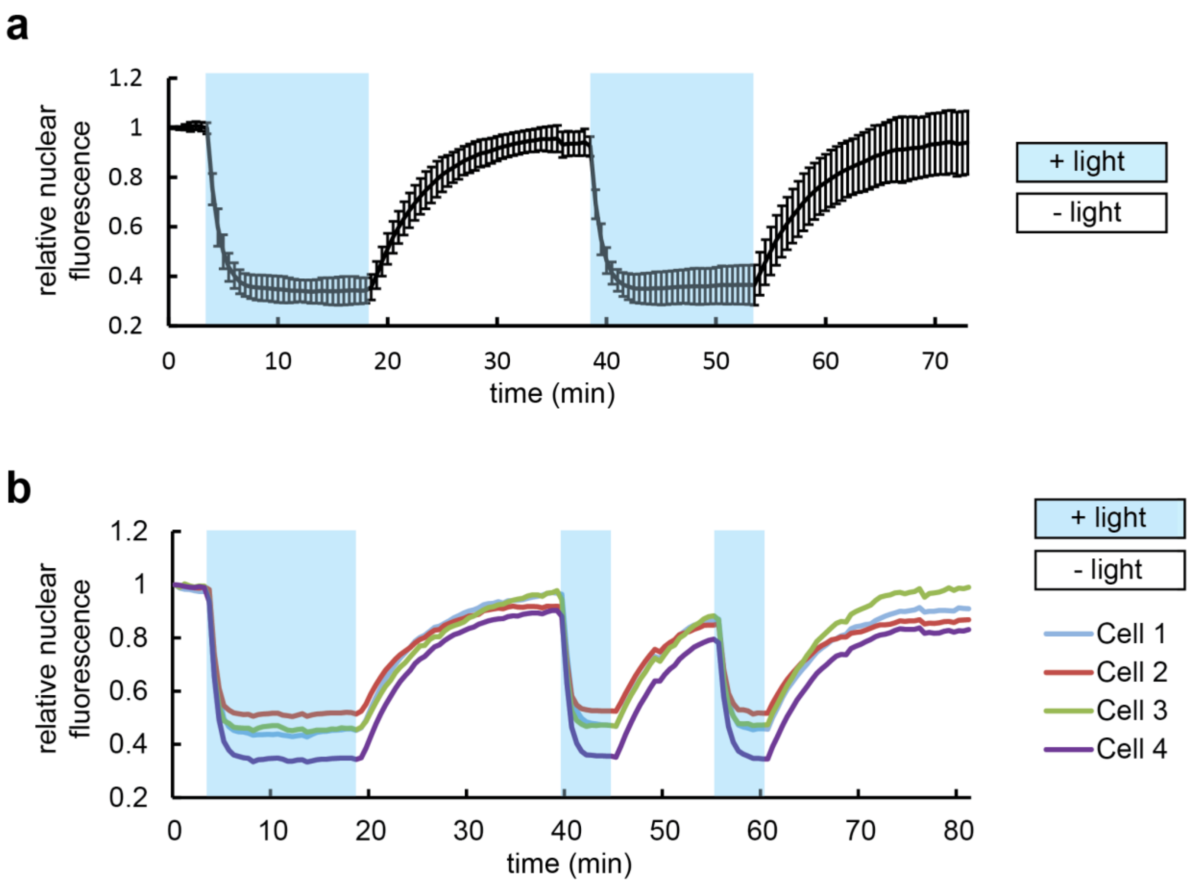

Supplementary Figure 5 | Nuclear export can be induced repeatedly. (**a**) HEK 293T expressing NLS-mCherry-LEXY were incubated for 3 min in the dark, followed by two cycles of 15 min irradiation with blue light (indicated by the cyan box) and 20 min recovery in the dark (mean ± SD, n = 10 cells, 2 independent experiments). (**b**) HELA cells expressing NLS-mCherry-LEXY were incubated for 3 min in the dark followed by blue light irradiation (indicated by the cyan box) and dark recovery phases as indicated. Single-cell traces from 4 individual cells derived from 2 independent experiments are shown.

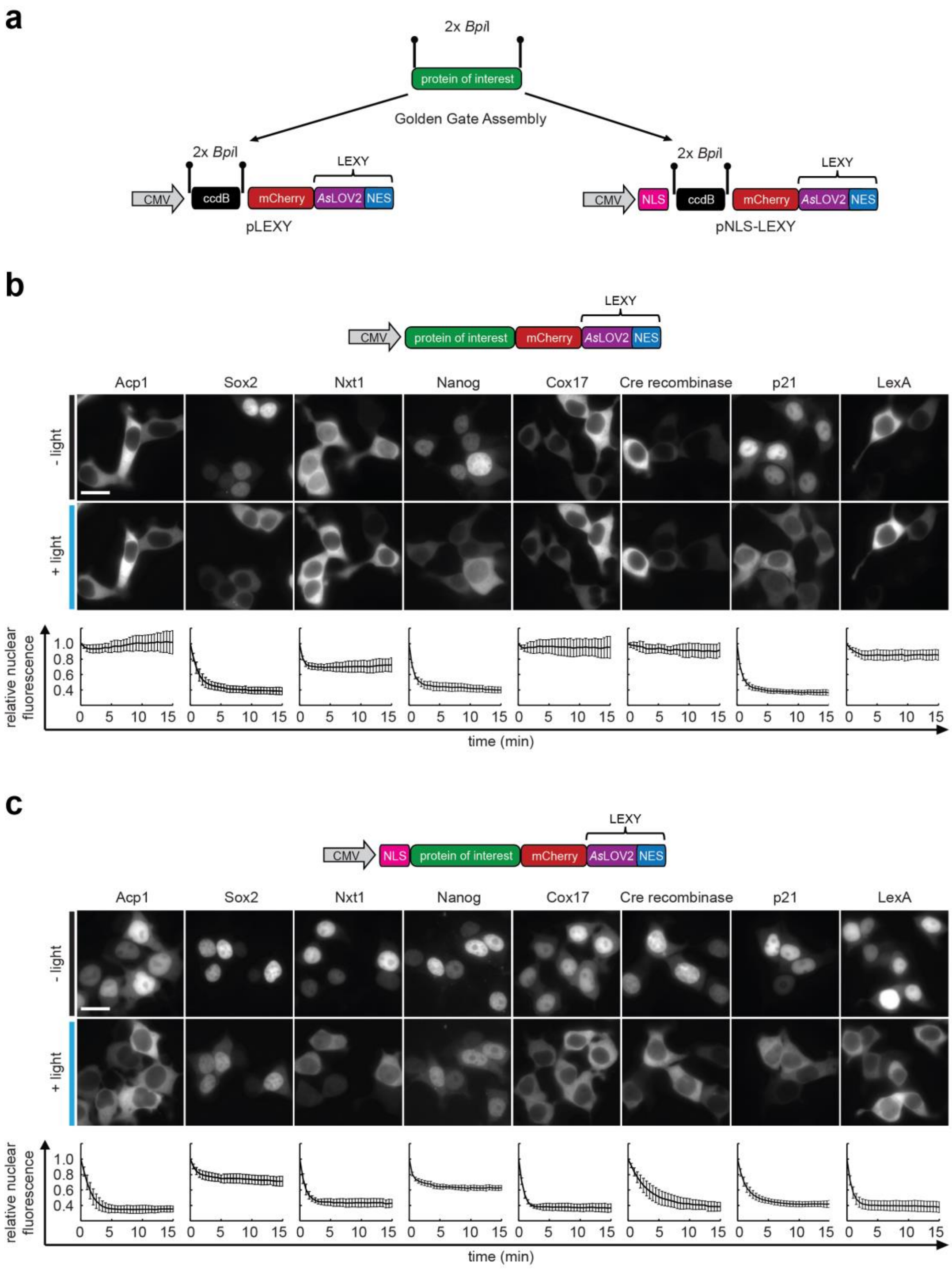

Supplementary Figure 6 | Proteins of different type, size and origin can be exported with LEXY. (**a**) Scheme showing golden gate cloning strategy enabling easy tagging of proteins with mCherry-LEXY using the two indicated entry

vectors. CMV, immediate early cycomegalovirus promoter; ccdB, death gene; NLS, cMyc^{P1A} NLS. (**b,c**) Top: Schematic of general construct used. Middle: Fluorescence microscopy images of 293T cells transfected with the indicated pLEXY (**b**) or pNLS-LEXY (**c**) variants prior to induction (- light) and after 15 min of illumination (+ light). Bottom: Corresponding quantifications of the relative nuclear fluorescence over time (mean ± SD; sample sizes (i.e. number of cells) are n = 7 for Nxt1, n = 8 for Acp1, Cre recombinase and LexA, n = 11 for Sox2 and p21, n = 10 for Nanog and Cox17 (in **b**) and n = 7 for Acp1, n = 8 for Sox2, Nxt1, Nanog and p21, n = 9 for Cox17 and LexA, n = 10 for Cre recombinase (in **c**)).

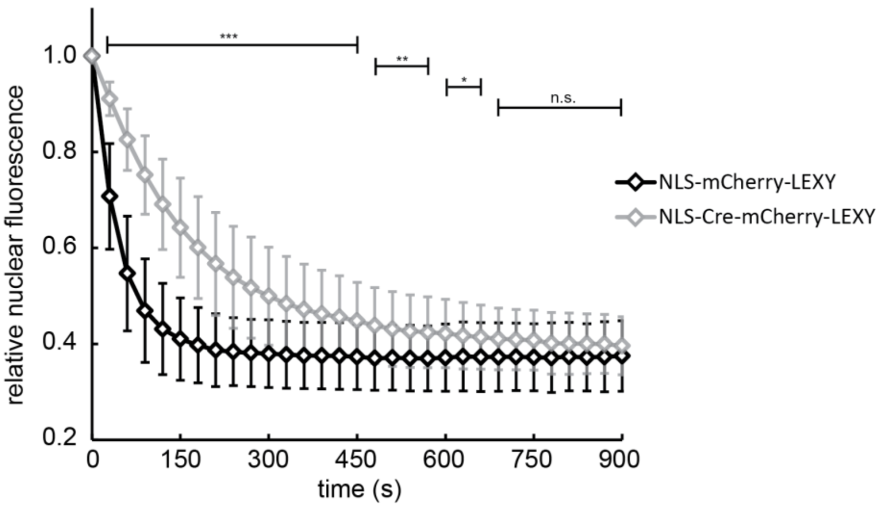

Supplementary Figure 7 | Effect of protein size and DNA-binding activity on export kinetics. 293T cells were transiently transfected with the indicated constructs and illuminated with blue light (mean ± SD, n = 22 cells from 3 independent experiments for NLS-mCherry-LEXY, and n = 33 cells from 4 independent experiments for NLS-Cre-mCherry-LEXY; $^{*}P < 0.05$, $^{**}P < 0.01$, $^{***}P < 0.001$ by Welch's *t*-test).

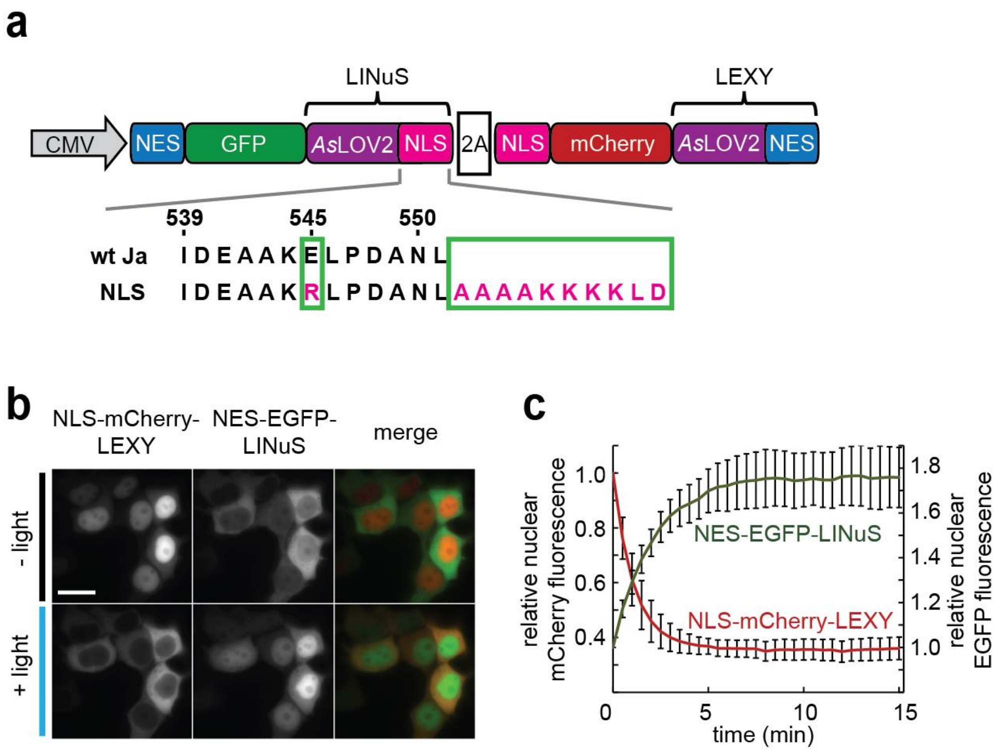

Supplementary Figure 8 | Simultaneous light-control of nuclear import and export. (**a**) Schematic of a construct for co-expression of NES-GFP-LINuS and NLS-mCherry-LEXY. CMV, immediate early cytomegalovirus promoter. NES (N-terminal and constitutive), IκBα NES. LINuS, light-inducible nuclear localization signal (biLINuS 2 variant)[2]. 2A, T2A peptide. Green boxes indicate amino acid residues in LINuS that are altered as compared to the wild type *As*LOV2 Jα helix (wt Jα). Numbers show the position of the corresponding residues in the full-length *As*LOV2 domain. (**b**) Fluorescence microscopy images of HEK 293T transiently transfected with the construct in (**a**) before (- light) and after (+ light) illumination with blue light for 15 min. Scale bar, 20 µm. (**c**) Corresponding quantification of the relative nuclear fluorescence of the cells shown in **b** (mean ± SD, n = 25 cells from 3 independent experiments).

Supplementary Tables

Supplementary Tab. 1: Plasmids used and developed in this study

#	Name	Backbone	Insert/expressed fusion protein	Promoter	NLS	*As*LOV2-NES hybrid	Source
**1**	pEGFP-N1	/	EGFP	CMV	/	/	Clontech
**2**	pcDNA3.1 (+)		/	CMV	/	/	Invitrogen
**3**	pmCherry-N1	/	mCherry	CMV	/	/	Clontech
**4**	H2B-GFP	pEGFP-N1	H2B-GFP	CMV	/	/	kind gift from Geoff Wahl[3] (Addgene plasmid #11680)
**5**	pDonor	/	ccdB	/	/	/	Invitrogen
**6**	pRL-TK	/	renilla luciferase	TK	/	/	Promega
**7**	pBluescript II SK	/	/	/	/	/	/
**8**	p53	/	wild type human p53	CMV	/	/	/
**9**	pDN34	pcDNA3.1 (-)	PKIt_NES-mCherry-AsLOV2-biLINuS2	CMV	AsLOV2-biNLS2	/	previous work by us[2] (Addgene plasmid #61343)
**10**	pDN77	pcDNA3.1 (-)	IkBα_NES-mCherry-AsLOV2-biLINuS2	CMV	AsLOV2-biNLS2	/	previous work by us[2] (Addgene plasmid #61347)
**11**	pDN100	pFR-Luc	firefly luciferase	4xLexA binding site dependent minimal promoter	/	/	previous work by us[2]
**12**	pDN101	pcDNA3.1 (-)	NLS-mCherry-AsLOV2-biNLS2	CMV	$cMyc^{P1A}$	/	This Work
**13**	pDN102	pcDNA3.1 (-)	NLS-mCherry-AsLOV2-NES	CMV	$cMyc^{P1A}$	NES 1	This Work
**14**	pDN103	pcDNA3.1 (-)	NLS-mCherry-AsLOV2-NES	CMV	$cMyc^{P1A}$	NES 2	This Work
**15**	pDN104	pcDNA3.1 (-)	NLS-mCherry-AsLOV2-NES	CMV	$cMyc^{P1A}$	NES 3	This Work
**16**	pDN105	pcDNA3.1 (-)	NLS-mCherry-AsLOV2-NES	CMV	$cMyc^{P1A}$	NES 4	This Work
**17**	pDN106	pcDNA3.1 (-)	NLS-mCherry-AsLOV2-NES	CMV	$cMyc^{P1A}$	NES 5	This Work
**18**	pDN107	pcDNA3.1 (-)	NLS-mCherry-AsLOV2-NES	CMV	$cMyc^{P1A}$	NES 6	This Work
**19**	pDN108	pcDNA3.1 (-)	NLS-mCherry-AsLOV2-NES	CMV	$cMyc^{P1A}$	NES 7	This Work
**20**	pDN109	pcDNA3.1 (-)	NLS-mCherry-AsLOV2-NES	CMV	$cMyc^{P1A}$	NES 8	This Work
**21**	pDN110	pcDNA3.1 (-)	NLS-mCherry-AsLOV2-NES	CMV	$cMyc^{P1A}$	NES 9	This Work
**22**	pDN111	pcDNA3.1 (-)	NLS-mCherry-AsLOV2-NES	CMV	$cMyc^{P1A}$	NES 10	This Work
**23**	pDN112	pcDNA3.1 (-)	NLS-mCherry-AsLOV2-NES	CMV	$cMyc^{P1A}$	NES 11	This Work
**24**	pDN113	pcDNA3.1 (-)	NLS-mCherry-AsLOV2-NES	CMV	$cMyc^{P1A}$	NES 12	This Work

Supplementary Tab. 1 continued

#	Name	Backbone	Insert/expressed fusion protein	Promoter	NLS	*As*LOV2-NES hybrid	Source
**25**	pDN114	pcDNA3.1 (-)	NLS-mCherry-AsLOV2-NES	CMV	$cMyc^{P1A}$	NES 13	This Work
**26**	pDN115	pcDNA3.1 (-)	NLS-mCherry-AsLOV2-NES	CMV	$cMyc^{P1A}$	NES 14	This Work
**27**	pDN116	pcDNA3.1 (-)	NLS-mCherry-AsLOV2-NES	CMV	$cMyc^{P1A}$	NES 15	This Work
**28**	pDN117	pcDNA3.1 (-)	NLS-mCherry-AsLOV2-NES	CMV	$cMyc^{P1A}$	NES 16	This Work
**29**	pDN118	pcDNA3.1 (-)	NLS-mCherry-AsLOV2-NES	CMV	$cMyc^{P1A}$	NES 17	This Work
**30**	pDN119	pcDNA3.1 (-)	NLS-mCherry-AsLOV2-NES	CMV	$cMyc^{P1A}$	NES 18	This Work
**31**	pDN120	pcDNA3.1 (-)	NLS-mCherry-AsLOV2-NES	CMV	$cMyc^{P1A}$	NES 19	This Work
**32**	pDN121	pcDNA3.1 (-)	NLS-mCherry-AsLOV2-NES	CMV	$cMyc^{P1A}$	NES 20	This Work
**33**	pDN122	pcDNA3.1 (-)	NLS-mCherry-AsLOV2-NES	CMV	$cMyc^{P1A}$	NES 21	This Work
**34**	pDN123	pcDNA3.1 (-)	NLS-mCherry-AsLOV2-NES	CMV	$cMyc^{P1A}$	NES 22	This Work
**35**	pDN124	pcDNA3.1 (-)	NLS-mCherry-AsLOV2-NES	CMV	$cMyc^{P1A}$	NES 23	This Work
**36**	pDN125	pcDNA3.1 (-)	NLS-mCherry-AsLOV2-NES	CMV	$cMyc^{P1A}$	NES 24	This Work
**37**	pDN126	pcDNA3.1 (-)	NLS-mCherry-AsLOV2-NES	CMV	$cMyc^{P1A}$	NES 25	This Work
**38**	pDN127	pcDNA3.1 (-)	NLS-mCherry-AsLOV2-NES	CMV	$cMyc^{P1A}$	NES 26	This Work
**39**	pDN128	pcDNA3.1 (-)	NLS-mCherry-AsLOV2-NES	CMV	$cMyc^{P1A}$	NES 27	This Work
**40**	pDN129	pcDNA3.1 (-)	NLS-mCherry-AsLOV2-NES	CMV	$cMyc^{P1A}$	NES 28	This Work
**41**	pDN130	pcDNA3.1 (-)	NLS-mCherry-AsLOV2-NES	CMV	$cMyc^{P1A}$	NES 29	This Work
**42**	pDN131	pcDNA3.1 (-)	NLS-mCherry-AsLOV2-NES	CMV	$cMyc^{P1A}$	NES 30	This Work
**43**	pDN132	pcDNA3.1 (-)	NLS-mCherry-AsLOV2-NES	CMV	$cMyc^{P1A}$	NES 31	This Work
**44**	pDN133	pcDNA3.1 (-)	NLS-mCherry-AsLOV2-NES	CMV	$cMyc^{P1A}$	NES 32	This Work
**45**	pDN134	pcDNA3.1 (-)	NLS-mCherry-AsLOV2-NES	CMV	$cMyc^{P1A}$	NES 33	This Work
**46**	pDN135	pcDNA3.1 (-)	NLS-mCherry-(wild-type)AsLOV2	CMV	$cMyc^{P1A}$	/	This Work
**47**	pDN136	pcDNA3.1 (-)	H2B-GFP-T2A-NLS-mCherry-AsLOV2-NES	CMV	$cMyc^{P1A}$	NES 21	This Work
**48**	pDN137 (pLEXY)	pEGFP-N1	*Bbs*I x ccdB x *Bbs*I-mCherry-LEXY	CMV	/	NES 21	This Work
**49**	pDN138 (pNLS-LEXY)	pEGFP-N1	NLS-*Bbs*IxccdBx*Bbs*I-mCherry-LEXY	CMV	$cMyc^{P1A}$	NES 21	This Work
**50**	pDN139	pEGFP-N1	NLS-ACP1-mCherry-AsLOV2-NES	CMV	$cMyc^{P1A}$	NES 21	This Work
**51**	pDN140	pEGFP-N1	NLS-Sox2-mCherry-AsLOV2-NES	CMV	$cMyc^{P1A}$	NES 21	This Work
**52**	pDN141	pEGFP-N1	NLS-Nanog-mCherry-AsLOV2-NES	CMV	$cMyc^{P1A}$	NES 21	This Work
**53**	pDN142	pEGFP-N1	NLS-Nxt1-mCherry-AsLOV2-NES	CMV	$cMyc^{P1A}$	NES 21	This Work
**54**	pDN143	pEGFP-N1	NLS-Cox17-mCherry-AsLOV2-NES	CMV	$cMyc^{P1A}$	NES 21	This Work
**55**	pDN144	pEGFP-N1	NLS-p21-mCherry-AsLOV2-NES	CMV	$cMyc^{P1A}$	NES 21	This Work
**56**	pDN145	pEGFP-N1	NLS-Cre_recombinase-mCherry-AsLOV2-NES	CMV	$cMyc^{P1A}$	NES 21	This Work
**57**	pDN146	pEGFP-N1	NLS-LexA-mCherry-AsLOV2-NES	CMV	$cMyc^{P1A}$	NES 21	This Work

Supplementary Tab. 1 continued

#	Name	Backbone	Insert/expressed fusion protein	Promoter	NLS	*As*LOV2 -NES hybrid	Source
**58**	pDN147	pEGFP-N1	ACP1-mCherry-AsLOV2-NES	CMV	not present	NES 21	This Work
**59**	pDN148	pEGFP-N1	Sox2-mCherry-AsLOV2-NES	CMV	not present	NES 21	This Work
**60**	pDN149	pEGFP-N1	Nanog-mCherry-AsLOV2-NES	CMV	not present	NES 21	This Work
**61**	pDN150	pEGFP-N1	Nxt1-mCherry-AsLOV2-NES	CMV	not present	NES 21	This Work
**62**	pDN151	pEGFP-N1	Cox17-mCherry-AsLOV2-NES	CMV	not present	NES 21	This Work
**63**	pDN152	pEGFP-N1	p21-mCherry-AsLOV2-NES	CMV	not present	NES 21	This Work
**64**	pDN153	pEGFP-N1	Cre_recombinase-mCherry-AsLOV2-NES	CMV	not present	NES 21	This Work
**65**	pDN154	pEGFP-N1	LexA-mCherry-AsLOV2-NES	CMV	not present	NES 21	This Work
**66**	pDN155	pcDNA3.1 (-)	IkBα_NES-EGFP-biLINuS2	CMV	as indicated	/	This Work
**67**	pDN156	pcDNA3.1 (-)	IkBα_NES-EGFP-biLINuS2-T2A-cMyc(P1A)_NLS-mCherry-LEXY	CMV	as indicated	as indicated	This Work
**68**	pDN157	pEGFP-N1	H2B-GFP-LEXY	CMV	/	NES 21	This Work
**69**	pDN158	pEGFP-N1	H2B-GFP-AsLOV2-NES27	CMV	/	NES 27	This Work
**70**	pDN159	pEGFP-N1	H2B-GFP-(wild-type)AsLOV2	CMV	/	/	This Work
**71**	pDN160	pcDNA3.1 (-)	NLS-mCherry-NES 17	CMV	$cMyc^{P1A}$	/	This Work
**72**	pDN161 (pANY-entry)	pEGFP-N1	*Bbs*I x ccdB x *Bbs*I	CMV	/	/	This Work
**73**	pDN162	pEGFP-N1	LexATF-T2A-NLS-LexA-mCherry-LEXY	CMV	$cMyc^{P1A}$	NES 21	This Work
**74**	pDN163	pEGFP-N1	LexATF-T2A-NLS-LexA-KRAB-mCherry-LEXY	CMV	$cMyc^{P1A}$	NES 21	This Work
**75**	pDN164	pEGFP-N1	LexATF-T2A-NLS-mCherry-LEXY	CMV	$cMyc^{P1A}$	NES 21	This Work
**76**	pPW1	pEGFP-N1	NLS-p53-mCherry-LEXY	CMV	$cMyc^{P1A}$	NES 21	This Work
**77**	pPW2	pcDNA3.1 (+)	p53-mCherry	CMV	/	/	This Work
**78**	pPW3	pcDNA3.1 (+)	p53-NES-mCherry	CMV	/	/	This Work

Supplementary Tab. 2: Oligonucleotide sequences

oligo #	Sequence 5' --> 3'	NES introduced
1	TTTTCGTCTCTGTGAGCAAGGGCGAGGAGGATAAC	/
2	TTTTCGTCTCTCATGCTAGCCAGCTTGGGTCTCCCTATAGTG	
3	CATGGCCGCAGCAAAGCGCGTGAAGCTGGAC	
4	TCACGTCCAGCTTCACGCGCTTTGCTGCGGC	
5	TTTTCGTCTCTCAATATTTTCTGCAGTTTTCTTAATCAG	
6	TTTTCGTCTCTTAGCTCGAGCGGCCGCCACTGTGCTGGATATC	
7	ATTGACCTGGCACTGAAGGAGCTGCCCGACCTGAACCTG	NES1
8	GCTACAGGTTCAGGTCGGGCAGCTCCTTCAGTGCCAGGT	
9	ATTGACCTGGCACTGAAGGAGCTGCCCGACCTGGACCTGGAC	NES2
10	GCTAGTCCAGGTCCAGGTCGGGCAGCTCCTTCAGTGCCAGGT	
11	ATTGACGAGCTGCAGAAGGAGCTGCCCGACCTGAACCTG	NES3
12	GCTACAGGTTCAGGTCGGGCAGCTCCTTCTGCAGCTCGT	
13	ATTGACGAGATGGTGAAGGAGCTGCCCGACATCCGCCTG	NES4
14	GCTACAGGCGGATGTCGGGCAGCTCCTTCACCATCTCGT	
15	ATTGACGAGATGGTGAAGGAGCTGCCCGACATCAACCTG	NES5
16	GCTACAGGTTGATGTCGGGCAGCTCCTTCACCATCTCGT	
17	ATTGACGAGCTGGTGAAGGAGCTGCCCGACATCCGCCTG	NES6
18	GCTACAGGCGGATGTCGGGCAGCTCCTTCACCAGCTCGT	
19	ATTGACGAGATCGCAAAGGAGCTGCCCGACCTGAACCTGGAGAACCTGCAG	NES7
20	GCTACTGCAGGTTCTCCAGGTTCAGGTCGGGCAGCTCCTTTGCGATCTCGT	
21	ATTGACGAGCTGGCAAAGGAGCTGCCCGACCTGAACCTGGAC	NES8
22	GCTAGTCCAGGTTCAGGTCGGGCAGCTCCTTTGCCAGCTCGT	
23	ATTGACGAGATCGCAAAGGAGCTGCCCGACGTGAACCTGGAC	NES9
24	GCTAGTCCAGGTTCACGTCGGGCAGCTCCTTTGCGATCTCGT	
25	ATTGACGAGATCGCAAAGGAGCTGCCCGACCTGAACCTGGAC	NES10
26	GCTAGTCCAGGTTCAGGTCGGGCAGCTCCTTTGCGATCTCGT	
27	ATTGACGAGCTGGCCAAGGAGCTGCCCGACCTGAACCTGGACAGC	NES11
28	GCTAGCTGTCCAGGTTCAGGTCGGGCAGCTCCTTGGCCAGCTCGT	
29	ATTGACGAGCTGGCCAAGGAGCTGCCCGACCTGAACCTGGACAGCAGC	NES12
30	GCTAGCTGCTGTCCAGGTTCAGGTCGGGCAGCTCCTTGGCCAGCTCGT	
31	ATTGACGAGCTGGCCAAGGAGCTGCCCGACCTGAACCTGGACAGCAGCGAC CTGAGC	NES13
32	GCTAGCTCAGGTCGCTGCTGTCCAGGTTCAGGTCGGGCAGCTCCTTGGCCAG CTCGT	
33	ATTGAGGAGCTGGCCAAGGAGCTGCCCGACCTGAACCTGGAC	NES14
34	GCTAGTCCAGGTTCAGGTCGGGCAGCTCCTTGGCCAGCTCCT	
35	ATTGACGAGCTGCTGAAGGAGCTGCCCGACCTGAACCTGGAC	NES15
36	GCTAGTCCAGGTTCAGGTCGGGCAGCTCCTTCAGCAGCTCGT	

Supplementary Tab. 2 continued

oligo #	Sequence 5' --> 3'	NES introduced
37	ATTGACGAGCTGGCCAAGCTGCTGCCCGACCTGAACCTGGAC	NES16
38	GCTAGTCCAGGTTCAGGTCGGGCAGCAGCTTGGCCAGCTCGT	
39	ATTGACGAGCTGGCCAAGGCCCTGCCCGACCTGAACCTGGAC	NES17
40	GCTAGTCCAGGTTCAGGTCGGGCAGGGCCTTGGCCAGCTCGT	
41	ATTGACGAGCTGGCCAAGGAGCTGGAGGACCTGAACCTGGAC	NES18
42	GCTAGTCCAGGTTCAGGTCCTCCAGCTCCTTGGCCAGCTCGT	
43	ATTGACGAGCTGGCCAAGGAGCTGGCCGACCTGAACCTGGAC	NES19
44	GCTAGTCCAGGTTCAGGTCGGCCAGCTCCTTGGCCAGCTCGT	
45	ATTGACGAGCTGGCCAAGGAGCTGCCCGACCTGGACCTGGAC	NES20
46	GCTAGTCCAGGTCCAGGTCGGGCAGCTCCTTGGCCAGCTCGT	
47	ATTGACGAGCTGCTGAAGGAGCTGGCCGACCTGAACCTGGAC	NES21
48	GCTAGTCCAGGTTCAGGTCGGCCAGCTCCTTCAGCAGCTCGT	
49	ATTGACGAGCTGGCCAAGGAGCTGGCCGACCTGGACCTGGAC	NES22
50	GCTAGTCCAGGTCCAGGTCGGCCAGCTCCTTGGCCAGCTCGT	
51	ATTGACGAGCTGGCCAAGCTGCTGGAGGACCTGGACCTGGAC	NES23
52	GCTAGTCCAGGTCCAGGTCCTCCAGCAGCTTGGCCAGCTCGT	
53	ATTGACGAGCTGGCCAAGCTGCTGGCCGACCTGGACCTGGAC	NES24
54	GCTAGTCCAGGTCCAGGTCGGCCAGCAGCTTGGCCAGCTCGT	
55	ATTGAGGAGCTGGCCAAGCTGCTGCCCGACCTGAACCTGGAC	NES25
56	GCTAGTCCAGGTTCAGGTCGGGCAGCAGCTTGGCCAGCTCCT	
57	ATTGAGGAGCTGGCCAAGGCCCTGCCCGACCTGAACCTGGAC	NES26
58	GCTAGTCCAGGTTCAGGTCGGGCAGGGCCTTGGCCAGCTCCT	
59	ATTGAGGAGCTGGCCAAGGAGCTGGCCGACCTGAACCTGGAC	NES27
60	GCTAGTCCAGGTTCAGGTCGGCCAGCTCCTTGGCCAGCTCCT	
61	ATTGACGAGCTGCTGAAGCTGCTGCCCGACCTGAACCTGGAC	NES28
62	GCTAGTCCAGGTTCAGGTCGGGCAGCAGCTTCAGCAGCTCGT	
63	ATTGACGAGCTGCTGAAGGCCCTGCCCGACCTGAACCTGGAC	NES29
64	GCTAGTCCAGGTTCAGGTCGGGCAGGGCCTTCAGCAGCTCGT	
65	ATTGACGAGCTGGCCAAGCTGCTGGCCGACCTGAACCTGGAC	NES30
66	GCTAGTCCAGGTTCAGGTCGGCCAGCAGCTTGGCCAGCTCGT	
67	ATTGACGAGCTGGCCAAGGCCCTGGCCGACCTGAACCTGGAC	NES31
68	GCTAGTCCAGGTTCAGGTCGGCCAGGGCCTTGGCCAGCTCGT	
69	ATTGACGAGCTGGCCAAGCTGCTGCCCGACCTGGACCTGGAC	NES32
70	GCTAGTCCAGGTCCAGGTCGGGCAGCAGCTTGGCCAGCTCGT	
71	ATTGACGAGCTGGCCAAGGCCCTGCCCGACCTGGACCTGGAC	NES33
72	GCTAGTCCAGGTCCAGGTCGGGCAGGGCCTTGGCCAGCTCGT	

Supplementary Tab. 2 continued

oligo #	Sequence 5' --> 3'	NES introduced
73	ATTGATGAGGCGGCAAAAGAACTTCCAGATGCTAATTTG	
74	GCTACAAATTAGCATCTGGAAGTTCTTTTGCCGCCTCAT	
75	TTTTCGTCTCTAACATGCGGTGACGTCGAGGAGAATCCTGGCCCAGGCGCCGCAGCAAAGCGCGTGAAGCTG	
76	TTTTCGTCTCTCATGCCAGAGCCAGCGAAGTCTGC	
77	TTTTCGTCTCTTGTTAGCAGACTTCCTCTGCCCTCTCCACTGCCCTTGTACAGCTCGTCCATGCCGAGAGTG	
78	TTTTCGTCTCGAATTCCGCCACCATGAAGTCTTCCGCGTGGATCCGGCTTAC	
79	TTTTCGTCTCGAATTCCGCCACCATGGCCGCAGCAAAGCGCGTGAAGCTGGACATGAAGTCTTCCGCGTGGATC	
80	TTTTCGTCTCTTCACTTGTCTTCACTGGCTGTGTATAAGGGAGCCTGAC	
81	TTTTCGTCTCTGTGAGCAAGGGCGAGGAGGATAACATG	
82	TTTTCGTCTCCTTTCTTCTGCATTACGGGGCCGTC	
83	AAAACGTCTCCGAAAACCATGGGCTGGGAGGCCTC	
84	TTTTCGTCTCTGGCCGCTCGAGCTAGTCCAGGTTCAGGTC	
85	TTTTGCTAGCATGCCCAGCACCCGGATCCAGCAGCAGCTGGGCCAGCTGACCCTGGAGAACCTGCAGGTGAGCAAGGGCGAGGAGCTGTTCAC	
86	TTTTGCGGCCGCCTACTTGTACAGCTCGTCCATG	/
87	TTTTCGTCTCTCGAGCTAGTCCAGGTTCAGGTCGGCCAGCTCCTTC	
88	TTTTCGTCTCTTGTTAGCAGACTTCCTCTGCCCTCTCCACTGCCGTCCAGCTTTTTCTTCTTGGCTGC	
89	TTTTCGTCTCTCTCGAGCGGCCGCCACTGTGCTGGATATC	
90	TTTTCGTCTCTGGTGGAAGTGAGGGAGTCATGCTGATTAAG	
91	TTTTCGTCTCTCACCTGAACCTCCAGATCCACCCTTGTACAGCTCGTCCATG	
92	GGACTCAGATCTCGAGCTCAAGC	
93	TAGAGTCGCGGCCGCTCGAGTCATTGTCTTCACTGGCTGTGTATAAGGGAG	
94	TTTTAACGTCTCACATGGAGGAGCCGCAGTCAGATCCTAG	
95	TTTTCGTCTCTTCACGTCTGAGTCAGGCCCTTCT	
96	TTTTAAGCTTATGGAGGAGCCGCAGTCAGATC	
97	TTTAGGATCCTCCACCTCCGTCTGAGTCAGGCCCTTCTG	
98	TTTTGGAGGATCCGTGAGCAAGGGCGAGGAGGATAACATGG	
99	TTTTGGAGGTGGAGGATCCATGTTAGCCTTGAAATTAGCAGGTCTTGATATCGTGAGCAAGGGCGAGGAGG	
100	TTTTCTCGAGTTACTTGTACAGCTCGTCCATGCCGCCGGTG	

Supplementary Note 1

Details on LEXY engineering by "Jα helix topping"

The idea behind LEXY is to photocage an NES in the Jα helix, so that it is exposed and able to interact with CRM1 receptors only in the lit state. The possibility to photocage short peptides in the Jα helix of the *As*LOV2 domain has been shown by several groups[2, 4-7]. In all these cases, the specific peptide to be caged was either directly appended to the end of a truncated *As*LOV2 Jα helix[2, 6, 7] or embedded within the helix[2, 4, 5], thereby replacing a continuous stretch of *As*LOV2 residues by the sequence to be caged. In contrast, we used the wild type *As*LOV2 Jα helix itself as starting scaffold and applied synthetic biology principles to progressively introduce known, NES-like features while highly preserving the native Jα helix composition. We term this approach "Jα helix topping" to signify that the Jα helix is "topped up" with a new characteristic (an NES in this case). To this end, we first investigated the distribution of non-charged, hydrophobic residues naturally present within the wild type *As*LOV2 Jα helix. We found that the wild type Jα helix carries a pattern of hydrophobic residues (A542-L546-A549-L551) matching that of the mammalian NES consensus sequence (***L-x***(2,3)-[LIVFM]-x(2,3)-L-x-[LI])[1] considerably well (Supplementary Fig. 1). Next, we introduced up to four point mutations into the Jα helix in order to adapt several helix residues – in particular A542 and A549 - to corresponding hydrophobic residues present in well-

studied NESs (HIV REV[8], IkBα[9], p53[10] or PKI[11] NES) or to preferred (i.e. frequent) amino acids as derived from a previously reported NES sequence alignment[1] of 58 experimentally validated NESs (Supplementary Fig. 1, NES sequence logo and NES 1-10). The resulting ten *As*LOV2-NES hybrids were screened by qualitatively investigating the nucleocytoplasmic translocation of NLS-mCherry-*As*LOV2-NES fusion constructs in human embryonic kidney (HEK 293T) cells upon pulsatile blue light irradiation using an epifluorescence microscope (Supplementary Fig. 2a,b; NES 1-10). We found modest, but significant light-induced nuclear export only for the construct harbouring NES 8, which contains four leucines representing an ideal NES consensus (Supplementary Figs. 1 and 2b).

Using NES 8 as lead candidate, we then constructed a second (optimization) library (Supplementary Figs. 1 and 2b; NES 11-33) by adapting single or multiple residues in NES 8 to preferred residues derived from the NES sequence alignment[1], while leaving the four leucines representing the NES consensus (L542, L546, L549 and L551) untouched. We also included three *As*LOV2-NES hybrids (NES11-13) carrying an elongated C-terminus.

Supplementary References

1. la Cour, T. et al. NESbase version 1.0: a database of nuclear export signals. *Nucleic Acids Res* **31**, 393-396 (2003).
2. Niopek, D. et al. Engineering light-inducible nuclear localization signals for precise spatiotemporal control of protein dynamics in living cells. *Nature communications* **5**, 4404, doi:10.1038/ncomms5404 (2014).
3. Kanda, T., Sullivan, K.F. & Wahl, G.M. Histone-GFP fusion protein enables sensitive analysis of chromosome dynamics in living mammalian cells. *Curr Biol* **8**, 377-385 (1998).
4. Lungu, O.I. et al. Designing photoswitchable peptides using the AsLOV2 domain. *Chemistry & biology* **19**, 507-517 (2012).
5. Yi, J.J., Wang, H., Vilela, M., Danuser, G. & Hahn, K.M. Manipulation of endogenous kinase activity in living cells using photoswitchable inhibitory peptides. *ACS Synth Biol* **3**, 788-795 (2014).
6. Strickland, D. et al. TULIPs: tunable, light-controlled interacting protein tags for cell biology. *Nature methods* **9**, 379-384 (2012).
7. Yumerefendi, H. et al. Control of Protein Activity and Cell Fate Specification via Light-Mediated Nuclear Translocation. *PloS one* **10**, e0128443, doi: 10.1371/journal.pone.0128443 (2015).
8. Fischer, U., Huber, J., Boelens, W.C., Mattaj, I.W. & Luhrmann, R. The HIV-1 Rev activation domain is a nuclear export signal that accesses an export pathway used by specific cellular RNAs. *Cell* **82**, 475-483 (1995).
9. Johnson, C., Van Antwerp, D. & Hope, T.J. An N-terminal nuclear export signal is required for the nucleocytoplasmic shuttling of IkappaBalpha. *EMBO J* **18**, 6682-6693 (1999).
10. Stommel, J.M. et al. A leucine-rich nuclear export signal in the p53 tetramerization domain: regulation of subcellular localization and p53 activity by NES masking. *EMBO J* **18**, 1660-1672 (1999).
11. Hauer, J.A., Barthe, P., Taylor, S.S., Parello, J. & Padilla, A. Two well-defined motifs in the cAMP-dependent protein kinase inhibitor (PKIalpha) correlate with inhibitory and nuclear export function. *Protein Sci* **8**, 545-553 (1999).
